# 3dRNAscore: a distance and torsion angle dependent evaluation function of 3D RNA structures

**DOI:** 10.1093/nar/gkv141

**Published:** 2015-02-24

**Authors:** Jian Wang, Yunjie Zhao, Chunyan Zhu, Yi Xiao

**Affiliations:** Biomolecular Physics and Modeling Group, Department of Physics and Key Laboratory of Molecular Biophysics of the Ministry of Education, Huazhong University of Science and Technology, Wuhan 430074, Hubei, China

## Abstract

Model evaluation is a necessary step for better prediction and design of 3D RNA structures. For proteins, this has been widely studied and the knowledge-based statistical potential has been proved to be one of effective ways to solve this problem. Currently, a few knowledge-based statistical potentials have also been proposed to evaluate predicted models of RNA tertiary structures. The benchmark tests showed that they can identify the native structures effectively but further improvements are needed to identify near-native structures and those with non-canonical base pairs. Here, we present a novel knowledge-based potential, 3dRNAscore, which combines distance-dependent and dihedral-dependent energies. The benchmarks on different testing datasets all show that 3dRNAscore are more efficient than existing evaluation methods in recognizing native state from a pool of near-native states of RNAs as well as in ranking near-native states of RNA models.

## INTRODUCTION

RNA molecules play different biological roles besides messengers between DNA and protein (1,2), e.g. regulatory functions (3). Like proteins, the 3D structural information is needed for better understanding of the functions of RNAs. Since the number of available RNA experimental structures is very limited at present, several computational methods have been proposed for structural modeling or RNA tertiary structures prediction (4–13). These methods usually generate a large set of candidates that need to be evaluated.

Knowledge-based statistical potential has been proved to be a powerful approach for evaluating models of protein tertiary structures (14–16). Currently, some knowledge-based potentials have also been proposed to evaluate models of RNA tertiary structures (17–21). For examples, the Ribonucleic Acids Statistical Potential (RASP) developed by Capriotti *et al*. (17,19) and the coarse-grained and all-atom RNA KB potentials by Bernauer *et al*. (18). The all-atom version of RASP, RASP-ALL, is a distance-dependent statistical potential with 23 clustered atom types and is trained on a non-redundant training set (randstr) generated by MODELLER (22). The RNA KB potentials are also distance-dependent statistical potentials. In the coarse-grained version of RNA KB potential, five atoms (P, C4′ in backbone, and C2, C4, C6 in base) are selected to represent the nucleotide. In the all-atom version of RNA KB potential, unlike RASP in which the atom types are clustered, the atom types in different nucleotides are considered to be different, so totally 85 atom types are considered rather than 23 atom types used in RASP-ALL. Furthermore, The RNA KB potentials used a Dirichlet process mixture model to obtain the distance distributions instead of bin counting (23). The fully differentiable feature also makes it possible for molecular dynamics simulations. The benchmark tests showed that the RASP and KB potentials could identify the native state structures effectively (17,18). However, further improvements are needed to rank near-native structures and pick out the structure closest to the native state from near-native structures including those with non-canonical base pairs, which is important in the prediction of RNA tertiary structures. Besides, there are other statistical potentials for evaluating RNA tertiary structures embedded in the RNA tertiary structure prediction programs and they have been compared in (17). For example, a full atom RNA potential (FARFAR, fragment assembly of RNA with full-atom refinement) available within the ROSETTA suite was successfully used for the de novo prediction and design of non-canonical RNA 3D structures (6,11). This full-atom potential contains weak carbon hydrogen bonding and solvation terms, as well as a complete description for potential hydrogen bonds between bases and backbone oxygen atoms.

In this work, we introduce a novel all-heavy-atom knowledge-based statistical potential, 3dRNAscore, to evaluate the 3D structure of RNA. Unlike the aforementioned two knowledge-based potentials that utilize the distances between atoms, a new energy contribution based on backbone torsion angle (dihedral) is involved in 3dRNAscore. The dihedral-based energy can describe the flexibility of RNA molecules more efficiently (24,25). Furthermore, we also consider the RNA stacking interactions in adjacent bases in the calculation of the distance-based energy of 3dRNAscore. It turns out that 3dRNAscore performs better than RASP and KB potentials in identifying RNA native structures from a pool of structural decoys as well as ranking a tremendous amount of near-native RNA tertiary structures.

## MATERIALS AND METHODS

**Figure 1.**
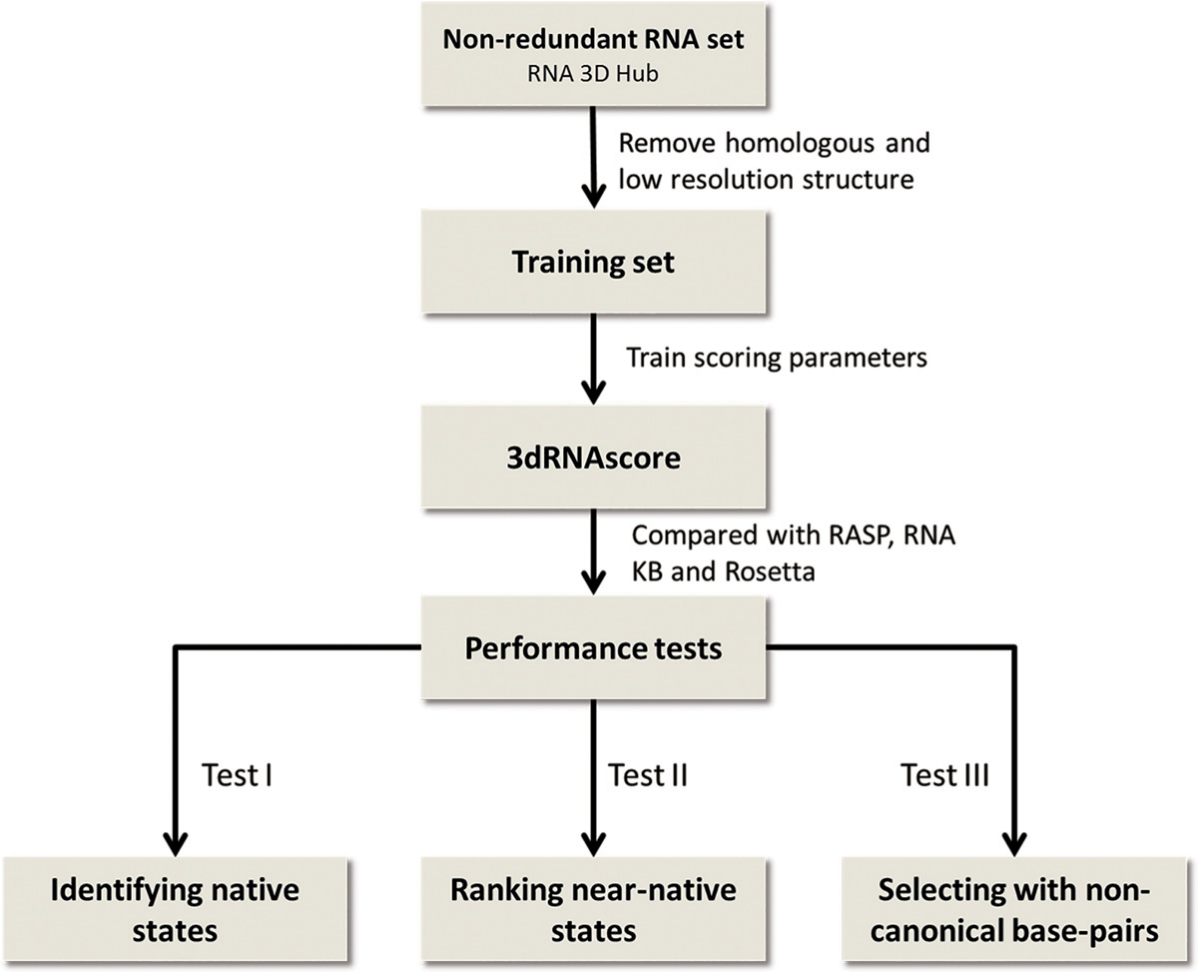
Flow charts of the building steps of 3dRNAscore.

The steps for building 3dRNAscore (Figure 1) are as follows. First, we design the functional form of 3dRNAscore from Boltzmann distribution, which contains two energy terms: the distance-dependent energy and the backbone dihedral-dependent energy. Second, in order to train the parameters in the scoring function, we select a training set of non-redundant RNA tertiary structures in which the structures having high similarity and those having similar sub-motifs with the structures in the test sets are removed. Using the training set, we determine the parameters in the scoring function. Third, we use the test sets to test the performance of 3dRNAscore. Here, we select three existing test sets. We use different metrics to compare the performance of 3dRNAscore with other scoring methods. The detail of each building step of 3dRNAscore is described in the following figure.

### Distance-dependent energy

Our knowledge-based potential 3dRNAscore is composed of two parts: the first part is based on distance between any two non-bonded heavy atoms located at different residues in the molecule, and the second part is based on the backbone torsion angles. Among them, the distance-dependent part is constructed as a mean force potential (26) derived from Boltzmann distribution.

Samudrala (27) pointed out that three assumptions underlie the mean force potential scoring method. The first assumption is that the total free energy of a molecule relative to some reference state, $\Delta G$, can be expressed as a sum of the relative free energy $\Delta G(R)$ of a number of individual contributions, where *R* represents the value of a ‘reaction coordinate’. The reaction coordinate may be any convenient measure of properties of the molecule. When using the distance *d* between atoms *i* and *j* of types *a* and *b* as the reaction coordinate, the assumption can be expressed as:

(1) \begin{equation*}
\Delta G = \Delta G(\{ d_{ab}^{ij} \} ) = \sum\limits_{ij} {\Delta G_{ij} (d_{ab}^{ij} } )
\end{equation*}

The second assumption is that the relative free energy can be deduced from the inverse of Boltzmann's law:

(2) \begin{equation*}
\Delta G(d) = - k_{\rm B} T{\rm ln}\frac{{f^{{\rm OBS} }(d)}}{{f^{{\rm REF}} (d)}}
\end{equation*}

Substituting Equation (2) into Equation (1), we have:

(3) \begin{equation*}
\Delta G = - k_{\rm B} T\sum\limits_{ij} {\ln \frac{{f_{ab}^{{\rm OBS}} (d_{ab}^{ij} )}}{{f_{ab}^{{\rm REF}} (d_{ab}^{ij} )}}}
\end{equation*}

where *T* is the absolute temperature and is set to 298 K, $k_{\rm B}$ is the Boltzmann's constant, $f_{ab}^{{\rm OBS}} (d_{ab}^{ij} )$ is the observed probability of the distance of $d_{ab}^{ij}$ between two atoms of types *a* and *b* in native RNA structures and $f_{ab}^{{\rm REF}} (d_{ab}^{ij} )$ is the probability of the distance between two atoms of *a* and *b* in reference state structures.

The third assumption is the thermodynamic hypothesis (28): the lowest free energy conformation represents the native state. The Equation (3) thus is useful for addressing the issue of ranking near-native structures.

The probability $f_{ab}^{{\rm OBS}} (d_{ab}^{ij} )$ could be evaluated with the number of occurrence observed in experimental structures:

(4) \begin{equation*}
f_{ab}^{{\rm OBS}} (d_{ab}^{ij} ) = \frac{{N_{ab} (d_{ab}^{ij} )}}{{\sum\limits_d {N_{ab} (d_{ab}^{ij} )} }} = \frac{{N_{ab} (d_{ab}^{ij} )}}{{N_{ab} }}
\end{equation*}

The probability $f_{ab}^{{\rm REF}} (d_{ab}^{ij} )$ could not be compiled from experimental structures directly. It relies on which reference state we choose. The RASP (17) and KB (18) potentials used averaged (RAPDF) (27) and quasi-chemical (KBP) (29) approximation reference states, respectively (30). These reference states consider covalent-bond constraints between atoms when counting the number of atom pair distances and usually need larger datasets of experimental RNA structures to obtain accurate potentials. In the average reference state, $f_{ab}^{{\rm REF}} (d_{ab}^{ij} )$ is evaluated by ignoring the type of atoms:

(5) \begin{equation*}
f_{ab}^{{\rm REF}} (d_{ab}^{ij} ) = \frac{{\sum\limits_{ab} {N_{ab} } (d_{ab}^{ij} )}}{{\sum\limits_{ab} {\sum\limits_d {N_{ab} } } (d_{ab}^{ij} )}} = \frac{{N(d_{ab}^{ij} )}}{N}
\end{equation*}

Equation (3) could then be expanded by substituting Equations (4) and (5) to it:

(6) \begin{eqnarray*}
&&\Delta G_{ij} (d_{ab}^{ij} ) = \nonumber \\
&& - k_{\rm B} T\ln \frac{{f_{ab}^{{\rm OBS}} (d_{ab}^{ij} )}}{{f_{ab}^{{\rm REF}} (d_{ab}^{ij} )}} = - k_{\rm B} T\ln \frac{{N_{ab} (d_{ab}^{ij} )N}}{{N(d_{ab}^{ij} )N_{ab} }}
\end{eqnarray*}

where $N_{ab} (d_{ab}^{ij} )$ is the counts of the occurrence of the distance of *d* between two atoms of types *a* and *b*. *N* is the total counts. $N(d_{ab}^{ij} )$ is the counts of the occurrence of the distance *d* regardless of atom types. $N_{ab}$ is the counts of the occurrence of atom pairs of types *a* and *b* in whole distance region.

In general, the atom pair in which the two atoms belong to two adjacent nucleotides along the sequence would not be considered. This is because that there may be bonding interactions between two atoms in adjacent nucleotides. The distance-dependent statistical potential just considers non-bonding interactions. However, atoms in adjacent bases do not have bonding interactions, and there exist base stacking interactions between them. Therefore, unlike other statistical potentials, the atom pair in two adjacent bases are considered in 3dRNAscore. Furthermore, the maximum value of the distance *d* in the process of statistics, namely the cutoff, is taken as 20 Å in 3dRNAsocre like other scoring methods.

Our all-heavy-atom distance-dependent potential utilizes all the 85 atom types (Table 1) in the four nucleotides: adenine (A), cytosine (C), guanine (G) and uracil (U). We number these 85 atom types from 1 to 85. For each atom-pair from 1–1 to 85–85, we count the distance distribution in a discrete space with a bin width of 0.15 Å. We use a matrix to represent the distance distribution information that each row denotes an atom-pair type and each column the counts of occurrences of the distance in the corresponding bin. So the data in the *i*th row and *j*th column of the matrix represents the counts of occurrences that the *i*th atom-pair has appeared at a distance of *j* in native RNA structure. Finally, we deposit the matrix into a parameter file that will be used in the scoring process.

**Table 1.** Atom types in 3dRNAscore potential

A	P OP1 OP2 O5′ C5′ C4′ O4′ C3′ O3′ C2′ O2′ C1′
	N9 C8 N7 C5 C6 N6 N1 C2 N3 C4
U	P OP1 OP2 O5′ C5′ C4′ O4′ C3′ O3′ C2′ O2′ C1′
	N1 C2 O2 N3 C4 O4 C5 C6
C	P OP1 OP2 O5′ C5′ C4′ O4′ C3′ O3′ C2′ O2′ C1′
	N1 C2 O2 N3 C4 N4 C5 C6
G	P OP1 OP2 O5′ C5′ C4′ O4′ C3′ O3′ C2′ O2′ C1′
	N9 C8 N7 C5 C6 O6 N1 C2 N2 N3 C4

### Selection of the bin width

The probability $f_{ij}^{{\rm OBS}} (d)$ and $f_{ij}^{{\rm REF}} (d)$ are stored as histograms with bin width of $\Delta d$. The size of the bin has a great influence on the probability distribution. Once the bin width is oversized, the probability $f_{ij}^{{\rm OBS}} (d)$ and $f_{ij}^{{\rm REF}} (d)$ stored would be truly rough. When the bin width is undersized, there may be none or little samples located in certain bins, leading to an inappropriate and artificial discontinuity of the probability distribution. The size of the bin width should be compatible with the total counts of the samples *N*. With the increasement of the number of the samples, the size of the bin should be as small as possible, the samples located in the bins should be sufficient, and the samples of the bins should be quite a very small part of the total samples.

Sippl (26) used a bin width of 1 Å, Samudrala (27) used a bin width of 1 Å and then carried out spline fitting, Capriotti (RASP) also used a bin width of 1 Å, Bernauer (KB) used a Dirichlet process mixture model, which leads to analytically differentiable potential functions, rather than fixed binning and spline fitting.

According to Scott's work (31) in 1979, the most appropriate bin width, which provides the most efficient and unbiased estimation of the population distribution, is achieved when:

(7) \begin{equation*}
W = 3.49\sigma N^{ - \frac{1}{3}}
\end{equation*}

Here, *W* means the bin width, *σ* means the standard deviation, and *N* means the number of samples.

We then extracted information from experimental structure, and then got the total samples *N* and calculated the standard deviation *σ*. Thus, we found that the most appropriate value of the bin width for 3dRNAscore is 0.3 Å. Bin width of 0.3 Å is small relative to those aforementioned, which is due to the large amount of samples in training set.

### The problem of sparse data

In mathematical statistics, a larger size of samples yields a more accurate population distribution. A too small size of samples is likely to result in severe uncertainty for population distribution. The accuracy of the distance distribution of a specific pair of atom types thus heavily depends on the size of the total observations on this pair of atom types. That is to say, the $N_{ij}$ in Equation (4) should not be far too small.

In 1990, Sippl developed a method to address the problem of small data sets (26). He approximated the genuine frequency *g_ij_*(*r*) by the sum of the total densities $f(r)$ and the statistical frequencies $f_{ij} (r)$:

(8) \begin{equation*}
g_{ij} (d) \approx \frac{1}{{1 + m\sigma }}f(d) + \frac{{m\sigma }}{{1 + m\sigma }}f_{ij} (d)
\end{equation*}

where *m* is actually $N_{ij}$, $\sigma$ represents a custom constant. In the limit $m \to \infty$, $g_{ij} (r)$ converges to $f_{ij} (r)$. In the limit $m \to 0$, $g_{ij} (r)$ converges to $f(r)$. Equation (8) ensures that the genuine frequency distribution $g_{ij} (r)$ resembles the total distribution $f(r)$ when encountering the situation of sparse data.

In order to avoid sparse data, we checked all the training parameters. There are totally about 5.25 × 10^8^ atom pairs in the dataset of 317 structures. Hence, there are averagely ∼1090 samples (5.25 × 10^8^/(85 × 85 × 20/0.3)) located in each distance bin. The atom distances are ignored when we are counting the total samples for each nucleotide pair type (one of 85×85 types). There are averagely 39 030 samples for each nucleotide pair type. Thus, both the average number of samples in each bin (∼1090) and the average number of samples in each nucleotide pair type (∼39 030) are large enough to avoid the sparse data problem. Furthermore, we do not use the statistics in the 0–3 Å regions where sparse data most likely appear. Instead, we utilize a penalty to substitute it. That is to say, once the distance between two atoms is <3 Å, it will give a penalty to the total energy score. In physical sense, the penalty corresponds to the Van der Waals exclusive force between two atoms. These analyses show that 3dRNAscore has no need of using the method proposed by Sippl to address the issue of sparse data.

### Dihedral-dependent energy

**Figure 2.**
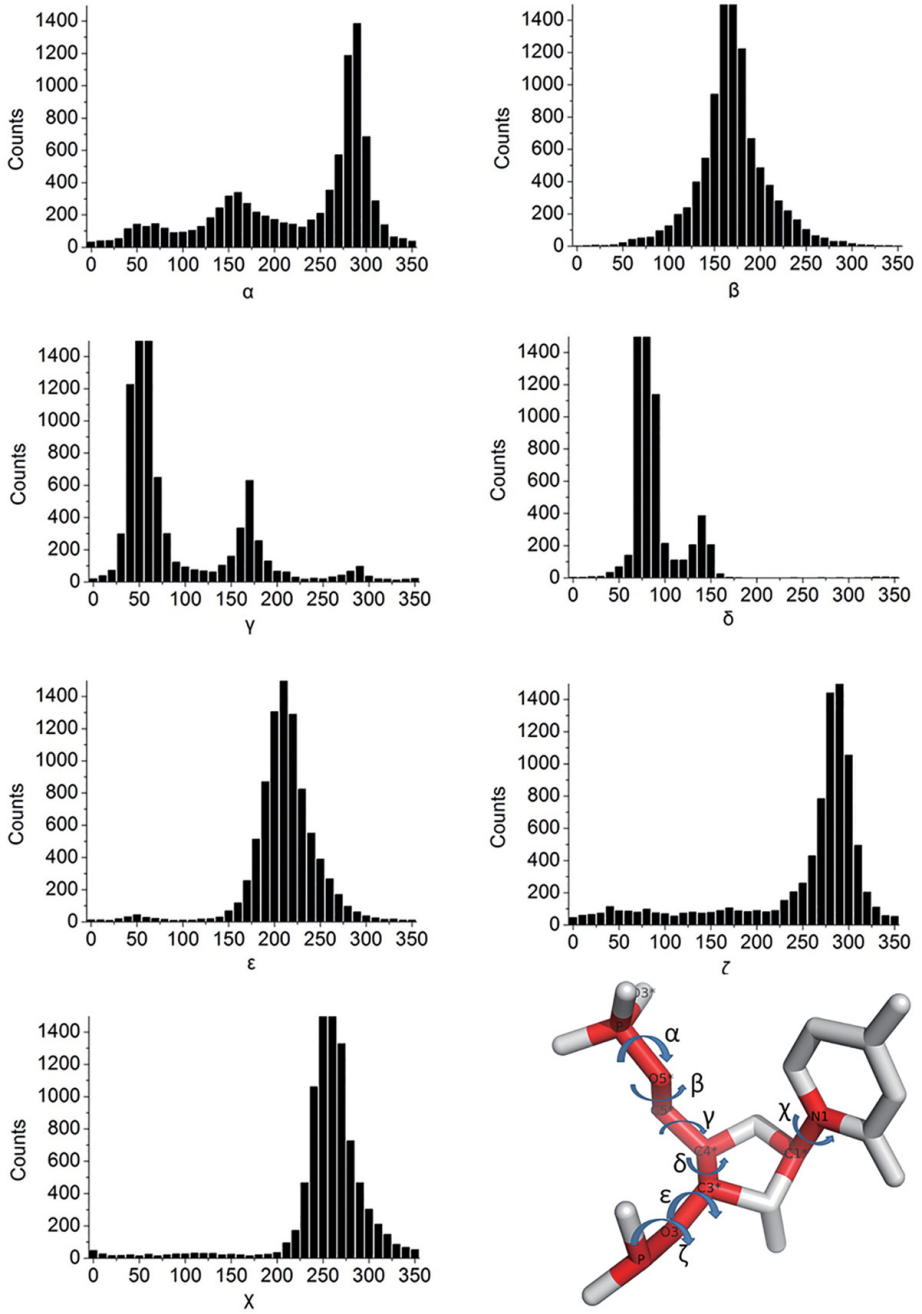
The frequency distributions of seven RNA torsion angles (α, β, γ, δ, ϵ, ζ for the nucleotide backbone, and χ for the base).

We used not only distance-dependent potential, but also a dihedral-dependent potential, involving seven RNA dihedral angles (α, β, γ, δ, ϵ, ζ for the nucleotide backbone, and χ for the base). First, we calculated their statistical distribution over the training set (Figure 2).

Once we get their statistical distributions, just like the distance-dependent potential, it can be assumed that all of these angles obey Boltzmann statistical distribution, and once again the mean force potential (26) is used:

(9) \begin{equation*}
\Delta G_i (\theta _a^i ) = - k_{\rm B} T\ln \frac{{f_a^{{\rm OBS}} (\theta _a^i )}}{{f_a^{{\rm REF}} (\theta _a^i )}}
\end{equation*}

where *T* is the absolute temperature that is set to 298 K, $k_{\rm B}$ is the Boltzmann's constant, $f_a^{{\rm OBS}} (\theta _a^i )$ is the observed probability of angle *i* (one of α, β, γ, δ, ϵ, ζ and χ) of *θ* degree (0–360) and type *a* in the database of experimental RNA structures and $f_a^{{\rm REF}} (\theta _a^i )$ is the expected probability of arbitrary angle *i* of *θ* degree and type *a* in reference state.

Then, we could also get a total score $\Delta G_{{\rm torsion}}$ in the same way as the distance-dependent potential energy:

(10) \begin{equation*}
f_i^{{\rm OBS}} (\theta ) = \frac{{N_i (\theta )}}{{\sum\limits_\theta {N_i (\theta )} }} = \frac{{N_i (\theta )}}{{N_i }}
\end{equation*}

(11) \begin{equation*}
f_i^{{\rm REF}} (\theta ) = \frac{{\sum\limits_i {N_i } (\theta )}}{{\sum\limits_i {\sum\limits_\theta {N_i } } (\theta )}} = \frac{{N(\theta )}}{N}
\end{equation*}

Just like the distance-dependent energy, we also use a matrix to store all the dihedral distribution information. Each row denotes a dihedral-pair type and each column the counts of occurrences of the dihedral value in the corresponding bin with a bin width of 4.5° according to eq.(7). So the matrix has 7 × 7 rows and 80 columns.

### Combination of the two energy terms

In 3dRNAscore the two energy terms are combined together to get the final total energy:

(12) \begin{equation*}
\Delta G_{{\rm total}} = \Delta G_{{\rm distance}} + \omega \Delta G_{{\rm dihedral}}
\end{equation*}

where $\Delta G_{{\rm total}}$ is the total energy, $\Delta G_{{\rm distance}}$ is the distance-dependent energy, $\Delta G_{{\rm dihedral}}$ is the dihedral-dependent energy, and $\omega$ is the weight.

To get an appropriate value for ω, we adopt a statistical optimization method, which is based on the coarse-grained force filed parameterization protocol in recent work by Leonarski *et al*. (32). First, we picked out four RNAs of different types from the training set. These four RNAs (PDB ID: 28SP, 1I9X, 1KPZ, 1J1U) are representative. 28SP is the structure of the most conserved internal loop in SRP RNA. It includes a hairpin loop and an internal loop, and the length is 28nt. 1I9X is the structure of a model branchpoint-U2 snRNA duplex. It is a duplex structure containing two bulge. It consists of 26 nucleotides. 1KPZ is the structure of a luteoviral P1–P2 frameshifting mRNA pseudoknot with the length of 33nt. 1J1U is a tRNA containing a multi-branch loop and it comprises 77 nucleotides. We use 3dRNA (12) to predict 1000 models for each RNA. We then calculated each model's DI and the energy score. After that, we use the gradient descent method to maximize the ES. The final optimized *ω* is 3.68.

### Training set

We have trained 3dRNAscore over the RNA 3D Hub non-redundant RNA set. RNA 3D Hub hosts non-redundant set of RNA-containing 3D structures extracted from experimental RNA structures according to the methodology described in Chapter 13 in ref. (33). Leontis’ group summarized two categories of structural redundancy existed in PDB/NDB database (34,35), which are redundancy within a given PDB file and redundancy in PDB/NDB database. They figured out ways to get rid of these redundancies, and then clustered all structures into 749 classes (Release 1.32, 2013–10–12) based on redundancies between structures. About how they get rid of these redundancies, please see Chapter 13 in (33). For each class, they chose a structure to represent it and assigned a unique and stable id to it. The dataset can be accessed at http://rna.bgsu.edu/nrlist/oldsite.html.

To construct the training set, we first gathered all the representative structures of 749 classes of RNA 3D Hub non-redundant RNA set. RNA 3D Hub ensured that sequence identity between any two sequences is <95%. By the way, RASP also used a sequence identity of 95% and the coverage of 80% to reduce redundancy in training set. RNA KB potential used a lower sequence identity of 80% to delete homologies in training set. We then used the blastn (36) program to discard all the RNAs with sequence identity >80% and coverage greater than 80%. After that, we carried out 3D structure alignment by the ARTS program (37) to discard all the RNAs where the coverage of aligned part was >80%. However, there exist recurrent motifs, e.g. the sarcin motifs and the GNRA motifs, in RNA 3D structures, and they often have quite different sequences and are hardly found by sequence alignment. These recurrent motifs may also affect the training of a scoring function and should remove this kind of redundancy. Based on RNA 3D Motif Atlas (38), which is a collection of RNA 3D motifs, we can find out the motifs contained in each structure in the training set. Then, RNAs having the motifs also contained in other RNAs in training set or in test sets were removed from the training set. These steps ensure that structures in the training set and test sets have no sequence and structural overlap. Finally, structures having low quality (resolution > 3.5 Å) are removed. Thus, there are 317 structures remained in the training set, which do not share any structures or motifs that are homologous to those in the test sets and have high structure quality (resolution < 3.5 Å).

### Test sets

We tested our knowledge-based potential using three different decoy sets available at present. Test set I is a randstr decoy set (17), which is generated by MODELLER (39) with a set of Gaussian restraints for dihedral angles and atom distances from 85 native structures. This is the largest decoy dataset in the benchmark. It can be downloaded from http://melolab.org/supmat.html.

Test set II consists of the decoys built by Bernauer group (18) and FARNA decoys (11). The former is generated by position restrained dynamics, REMD simulation and normal-mode perturbation method (40,41). In the REMD simulation, 1ns REMD simulations are performed for each RNA structures. The temperature is roughly distributed from 285 to 592 K for 50 different temperatures. In the normal-mode perturbation approach, the structures possess stereochemically correct bond lengths and angles but without correct contacts (42). These methods can generate decoy sets with RMSD ranging from 0 to 10 Å. It can be downloaded from http://csb.stanford.edu/rna. The FARNA decoys used in this study consist of lots of near-native tertiary models (11).

Test set III is the FARFAR decoy set which was generated by RNA modeling with ROSETTA-3.1 (11). The FARFAR decoy dataset consists of five lowest energy clusters of tertiary structures with non-canonical base pairs for each of 32 motifs. The FARNA and FARFAR decoys can be downloaded from http://www.stanford.edu/∼rhiju/data.html.

### Metrics of measuring RNA structures

To compare any two RNA structures quantitatively, we should make use of some metrics to assess their tertiary structures. The most commonly used metric is RMSD (root mean square deviation). RMSD depicts the global geometry differences between two RNA 3D structures but it is usually difficult to describe the hydrogen bond networks of RNA molecules. Hence, some metrics accounting for hydrogen-bonding networks intramolecular in RNA have been proposed to assess RNA structures. One of the commonly used metrics specifically devised for RNA is DI (deformation index) proposed by Parisien (43). The DI is defined as

(13) \begin{equation*}
{\rm DI}({\rm A},{\rm B}) = \frac{{{\rm RMSD}({\rm A},{\rm B})}}{{{\rm INF}({\rm A},{\rm B})}}
\end{equation*}

where RMSD reflects the geometry discrepancy and INF reflects the topology discrepancy. INF(A,B) is the interaction network fidelity between two structures A and B. Base-pairing and base-stacking are the two major interactions in RNA. These two interactions constitute the interaction network of RNA. Suppose $S_{\rm r}$ is the set of interactions in reference structure and $S_{\rm m}$ is the set of interactions in modeled structure, then the true positives TP is defined as ${\rm TP} = S_{\rm r} \cap S_{\rm m}$, the false positives FP is defined as ${\rm FP} = S_{\rm m} - S_{\rm r}$, and the false negatives FN is defined as ${\rm FN} = S_{\rm r} - S_{\rm m}$. In Parisien's paper, INF is defined as MCC, that is to say

(14) \begin{equation*}
{\rm INF}({\rm A},{\rm B}) = {\rm MCC}({\rm A},{\rm B})
\end{equation*}

where MCC is the Matthews correlation coefficient. They used the approximate definition of MCC (44,45):

(15) \begin{equation*}
{\rm MCC} \approx \sqrt {{\rm PPV} \times {\rm STY}}
\end{equation*}

where ${\rm PPV}({\rm precision}) = \frac{{{\rm TP}}}{{{\rm TP} + {\rm FP}}}$ and ${\rm STY}({\rm sensitivity}) = \frac{{{\rm TP}}}{{{\rm TP} + {\rm FN}}}$. For comparison, here we also use this approximation.

In this work, we use both RMSD and DI to measure how well a RNA model recapitulates the corresponding experimental structure in the benchmark of the performance of 3dRNAscore and other scoring functions.

## RESULTS

### Identifying native RNA structures

An important function of scoring methods is to identify the native-like tertiary structure of target RNA in a pool of structural decoys correctly. To compare such ability of 3dRNAscore with three existing RNA knowledge-based potentials: RASP (17), KB (18) and Rosetta (FARFAR) (6), for the sake of fairness, we use all-heavy-atom representation for all the potentials and evaluate them over the same decoy sets.

**Figure 3.**
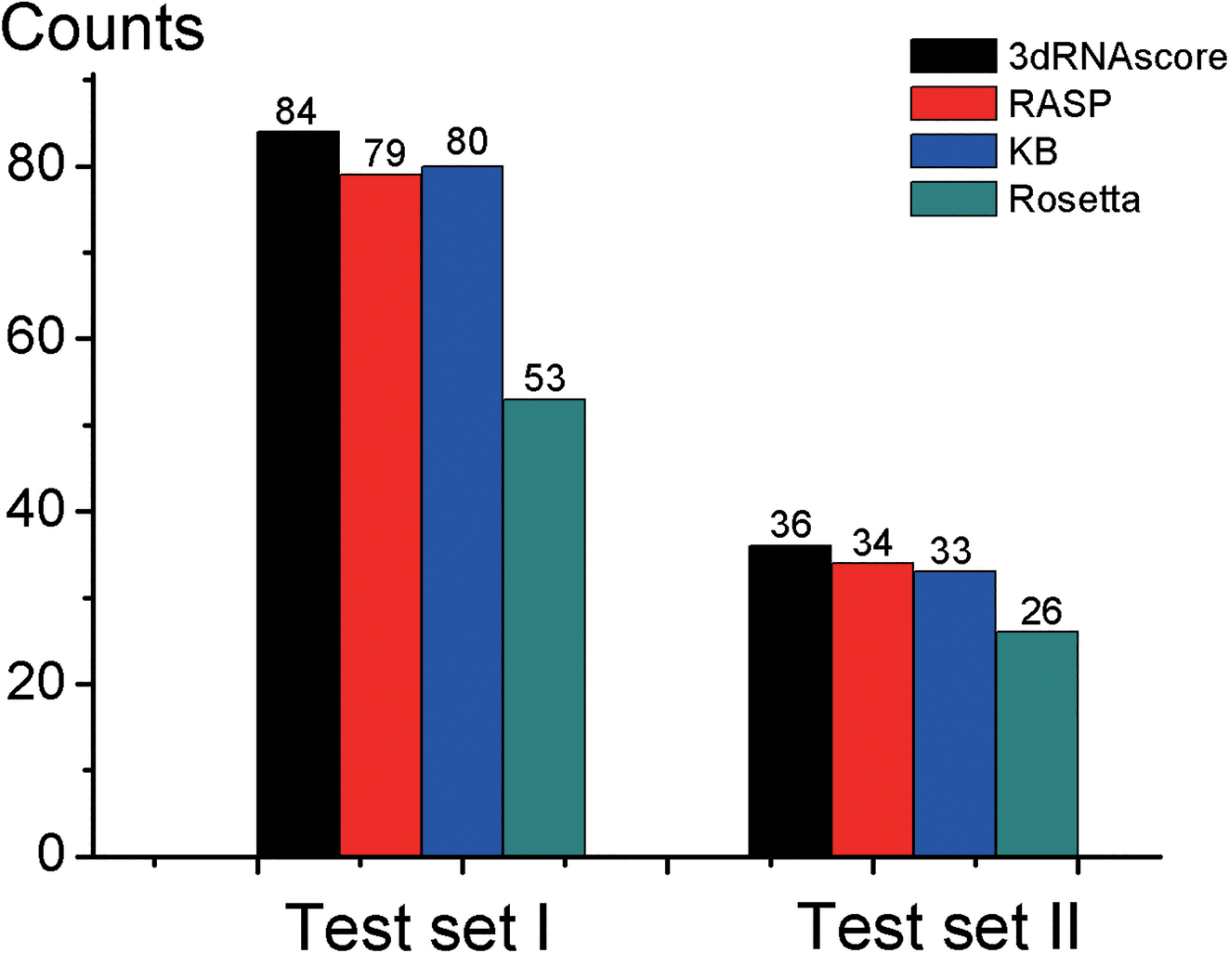
Counts of native states identified correctly from test set I and test set II by 3dRNAscore, RASP, KB and Rosetta, respectively.

Two tests are done on test set I and test set II, respectively (see Figure 3). When using 3dRNAscore, 84 out of 85 native structures are identified in test set I, and 36 out of 39 in test set II. For RASP, 79 out of 85 in test set I and 34 out of 39 in test set II. KB potentials could identify 80 out of 85 native structures in test set I, and 33 out of 39 in test set II. For Rosetta, the former is 53 out of 85 and the latter is 26 out of 39. These results show that 3dRNAscore has a better performance than other three methods on identifying native RNA structures.

It is noted that Capriotti's group has verified that RASP has a better performance than NAST (46) and the molecular force field energy function: AMBER pseudo-energies (47).

### Ranking near-native RNA structures

Another significant function of scoring function is to rank near-native structures reasonably. In the RNA structure prediction, a natural question is how to affirm that a predicted structure is closer than others to the native state. For RNA, a structure could be geometrically and topologically close to another structure. Hence, both RMSD and DI are used in this work to evaluate structure discrepancy. Mentioned earlier, RMSD measures similarity of two RNA structures from the aspect of geometry and DI is from both geometry and topology.

To describe the performance of a scoring function, the ES (enrichment score (18,48)) is employed here and it is defined as

(16) \begin{equation*}
{\rm ES} = \frac{{|E_{{\rm top}10\% } \cap R_{{\rm top}10\% } |}}{{0.1 \times 0.1 \times N_{{\rm decoys}} }}
\end{equation*}

where $E_{{\rm top}10\% }$ is the number of structures with energies (scores given by scoring function) in the lowest 10% of the energy range. For RMSD-based ES, *R*_top10%_ is the number of structures with RMSD in the lowest 10%. For DI-based ES, *R*_top10%_ is the number of structures with DI in the lowest 10%. $|E_{{\rm top}10\% } \cap R_{{\rm top}10\% } |$ is the intersection of $E_{{\rm top}10\% }$ and $R_{{\rm top}10\% }$. If the relationship between the scores and RMSD or DI is completely linear, then ES is equal to 10. If the relationship is random, ES is equal to 1, so

(17) \begin{equation*}
{\rm ES} = \left\{ {\begin{array}{*{20}c} {10,} & {{\rm perfect}\;{\rm scoring}} \\
{1,} & {{\rm perfectly}\;{\rm random}} \\
{ <1,} & {{\rm bad}\;{\rm scoring}} \\
\end{array}} \right.
\end{equation*}

We have benchmarked the performance of 3dRNAscore, RASP, KB and Rosetta in ranking near-native structures on test set II, using both RMSD and DI metrics. Table 2 shows that when we are using ES of DI, 3dRNAscore (ES = 4.5) outperforms other three scoring methods (RASP(ES = 3.8), KB(ES = 3.7) and Rosetta(ES = 2.7)) on the overall average level. It also outperforms them on the REMD decoys, normal mode decoys and FARNA decoys of test set II. The result is the same when the test set II is divided into two parts: NMR and X-ray. When we are using ES of RMSD, the result is similar. These results suggest that 3dRNAscore is better than other methods when it's used to rank near-native structures. A more detailed table than Table 2 and energy-RMSD (DI, INF) plots are provided in the supplementary data.

**Table 2.** Comparison of the performance of 3dRNAscore, KB potential and Rosetta methods in test set II

Decoy	RNA	Length	Method	Enrichment score (RMSD)				Enrichment score (DI)			
				3dRNAscore	KB	RASP	Rosetta	3dRNAscore	KB	RASP	Rosetta
Position restrained dynamics and REMD (A)	1duq	26	X-ray	8.5	7.6	7.6	7.1	8.3	7.5	7.6	7.0
	1f27	30	X-ray	8.3	7.9	6.6	6.2	8.1	7.8	6.6	6.2
	1msy	27	X-ray	7.5	5.7	5.7	3.6	7.6	6.0	5.6	3.5
	1nuj	24	X-ray	7.7	7.3	5.2	6.9	7.4	7.2	5.2	6.7
	434d	14	X-ray	8.0	7.7	7.0	6.8	7.6	7.7	6.9	6.8
Normal modes (B)	1duq	26	X-ray	7.5	7.0	5.7	3.8	6.9	7.0	5.7	3.5
	1esy	19	NMR	4.9	5.4	4.5	5.6	6.5	5.5	4.7	5.7
	1f27	30	X-ray	5.9	5.8	3.7	2.6	6.7	5.8	3.7	2.5
	1i9v	76	X-ray	6.1	2.6	5.3	3.0	5.5	2.7	5.1	3.0
	1kka	17	NMR	5.7	4.6	4.1	4.6	6.9	4.3	4.1	4.8
	1msy	27	X-ray	5.9	5.6	2.2	4.6	6.7	5.4	2.7	4.5
	1nuj	24	X-ray	7.1	7.4	5.9	2.4	4.5	7.0	5.9	2.2
	1qwa	21	NMR	3.5	3.2	2.0	3.8	3.7	3.3	2.2	3.9
	1×9k	62	X-ray	6.7	1.6	5.2	3.0	3.1	1.9	5.2	2.8
	1xjr	46	X-ray	7.7	5.4	7.9	2.2	6.2	5.0	7.9	2.5
	1ykq	19	X-ray	4.6	3.4	3.5	2.8	5.0	3.3	3.8	2.5
	1zih	12	NMR	7.7	5.4	5.7	6.6	6.9	5.3	5.7	6.5
	28sp	28	NMR	5.7	4.0	6.5	1.8	5.7	4.5	6.7	2.9
	2f88	34	NMR	6.8	5.4	4.9	4.4	4.1	5.4	4.7	4.2
	434d	14	X-ray	7.7	7.4	7.4	5.2	6.9	7.4	7.6	5.2
FARNA (C)	1a4d	41	NMR	2.4	3.8	2.0	0.8	2.1	4.0	2.0	0.7
	1csl	28	X-ray	2.0	1.5	1.6	1.3	2.4	1.4	1.6	1.4
	1dqf	19	X-ray	4.2	1.8	2.8	1.0	3.6	2.0	3.2	1.0
	1esy	19	NMR	3.2	3.7	4.8	1.2	3.8	3.5	4.2	1.1
	1i9x	26	X-ray	3.4	1.3	2.2	1.5	4.0	1.5	3.6	1.6
	1j6s	24	X-ray	0.2	1.4	0.2	0.6	1.6	1.7	0.8	0.5
	1kd5	22	X-ray	3.2	0.3	0.8	0.2	2.4	0.5	1.6	0.3
	1kka	17	NMR	1.4	1.2	0.6	0.6	2.0	0.8	0.8	0.6
	1l2x	27	X-ray	0.6	3.2	1.0	1.8	1.5	3.2	1.2	1.8
	1mhk	32	X-ray	1.6	1.2	1.6	1.0	1.4	1.2	1.4	1.2
	1q9a	27	X-ray	2.6	0.5	0.8	0.8	3.2	0.5	1.2	0.8
	1qwa	21	NMR	2.2	1.2	0.4	1.0	1.4	1.3	0.6	1.0
	1xjr	46	X-ray	2.4	2.0	2.4	1.2	3.2	1.9	3.4	1.0
	1zih	12	NMR	4.8	5.0	4.8	2	6.8	5.5	7.2	1.9
	255d	24	X-ray	2.4	0.7	0.6	1.3	2.0	0.7	0.6	1.1
	283d	24	X-ray	1.4	0.8	0.8	0.7	1.8	0.8	1.0	0.7
	28sp	28	NMR	2.8	1.5	3.0	1.7	3.8	1.2	4.2	1.8
	2a43	26	X-ray	2.2	2.0	1.4	0.6	3.2	1.8	2.0	0.6
	2f88	34	NMR	3.2	1.3	2.2	1.3	3.6	1.3	1.6	1.2
Average values		(A)		8.0	7.2	6.4	6.1	7.8	7.2	6.4	6.0
		(B)		6.2	4.9	5.0	3.8	5.7	4.9	5.0	3.8
		(C)		2.3	1.8	1.8	1.1	2.8	1.8	2.2	1.1
		X-ray		4.8	3.8	3.7	2.8	4.7	3.8	3.9	2.7
		NMR		4.2	3.5	3.5	2.7	4.4	3.5	3.7	2.8
		All		4.5	3.7	3.6	2.7	4.6	3.7	3.8	2.7

### Selecting correct structure containing non-canonical base pairs.

**Figure 4.**
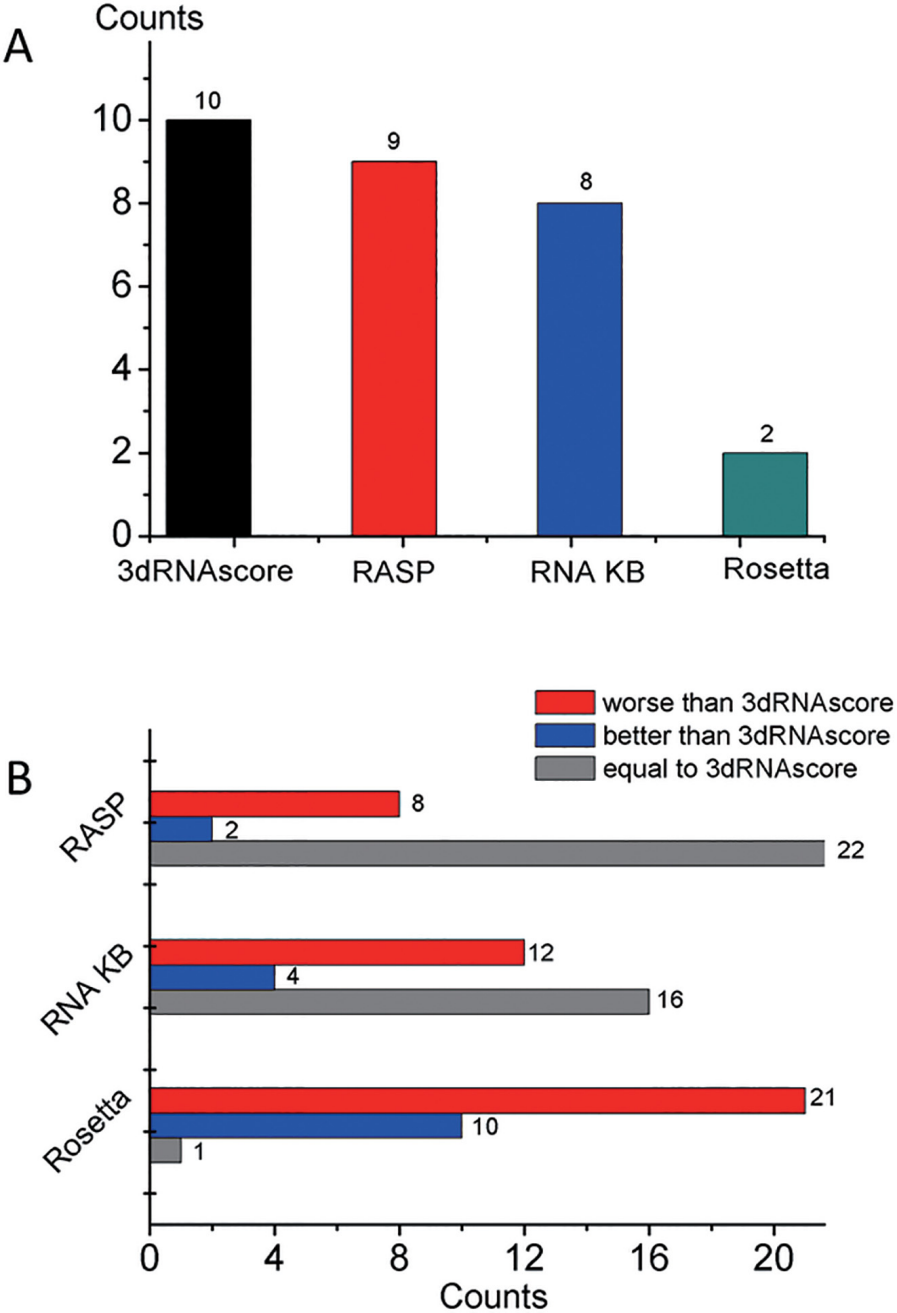
Results on test III. (**A**) The counts of models having the lowest energy selected by 3dRNAscore, RASP, RNA KB and Rosetta, respectively. (**B**) The red part represents the counts of models that are worse than those selected by 3dRNAscore. Blue represents the counts of models are that better than those selected by 3dRNAscore. Gray represents the counts of models that are the same as those selected by 3dRNAscore.

Non-canonical base pairs occur frequently in RNA tertiary folds and functional motifs (49–51). The selection of near-native conformations containing non-canonical base pairs is an inevitable and intractable problem in current RNA 3D structure prediction (6). Test set III is used to test the ability of 3dRNAscore in selecting correct structures containing non-canonical base pairs. Test set III (the FARFAR decoy set) consists of five lowest energy clusters of tertiary structures with non-canonical base pairs for each of 32 motifs. We calculated the energy score with RASP, KB, ROSETTAmin and 3dRNAscore, respectively. The lowest energy models according to each potential are then selected and compared with the lowest DI models. The results are presented in Table 4. 3dRNAscore, RASP, KB and Rosetta are able to identify the lowest DI models of 10, 9, 8 and 2 out of the 32 RNA motifs from the FARFAR decoy set, respectively (see Figure 4 (A)). 3dRNAscore can pick out models with lower DI than those by RASP, KB and Rosetta for 8, 12 and 21 out of 32 motifs and models with DI identical to those by RASP, KB and Rosetta for 22, 16 and 1 out of 32 motifs, respectively (see Figure 4 (B)). Moreover, 3dRNA can pick out models with DI greater than those by RASP, KB and Rosetta for 2, 4 and 10 out of 32 motifs, respectively.

**Table 3.** Comparison of performance of distance-dependent energy, dihedral-dependent energy, and total energy of 3dRNAscore in test set II

Decoy	RNA	Length	Method	Enrichment score (DI)		
				Distance term	Dihedral term	Total
Position restrained dynamics and REMD (A)	1duq	26	X-ray	8.2	7.5	8.3
	1f27	30	X-ray	7.9	5.7	8.1
	1msy	27	X-ray	7.4	6.7	7.6
	1nuj	24	X-ray	7.3	7.5	7.4
	434d	14	X-ray	7.5	7.0	7.6
Normal modes (B)	1duq	26	X-ray	6.3	4.8	6.9
	1esy	19	NMR	6.5	5.2	6.5
	1f27	30	X-ray	6.7	4.3	6.7
	1i9v	76	X-ray	5.5	4.6	5.5
	1kka	17	NMR	6.9	2.6	6.9
	1msy	27	X-ray	6.3	4.7	6.7
	1nuj	24	X-ray	4.5	1.6	4.5
	1qwa	21	NMR	3.5	2.2	3.7
	1×9k	62	X-ray	3.1	2.5	3.1
	1xjr	46	X-ray	5.8	4.8	6.2
	1ykq	19	X-ray	5.2	5.0	5.0
	1zih	12	NMR	5.9	3.4	6.9
	28sp	28	NMR	6.1	5.4	5.7
	2f88	34	NMR	3.9	3.2	4.1
	434d	14	X-ray	6.5	5.2	6.9
FARNA (C)	1a4d	41	NMR	1.8	2.4	2.1
	1csl	28	X-ray	2.2	1.8	2.4
	1dqf	19	X-ray	3.6	2.0	3.6
	1esy	19	NMR	3.8	2.4	3.8
	1i9x	26	X-ray	4.2	1.6	4.0
	1j6s	24	X-ray	0.6	2.2	1.6
	1kd5	22	X-ray	2.4	1.6	2.4
	1kka	17	NMR	1.7	0.8	2.0
	1l2x	27	X-ray	0.4	1.4	1.5
	1mhk	32	X-ray	1.2	0.6	1.4
	1q9a	27	X-ray	3.0	1.6	3.2
	1qwa	21	NMR	1.2	0.6	1.4
	1xjr	46	X-ray	3.0	1.8	3.2
	1zih	12	NMR	6.8	2.4	6.8
	255d	24	X-ray	1.8	0.8	2.0
	283d	24	X-ray	1.7	0.6	1.8
	28sp	28	NMR	3.6	4.2	3.8
	2a43	26	X-ray	2.8	1.4	3.2
	2f88	34	NMR	3.5	2.1	3.6
Average values		(A)		7.66	6.88	7.80
		(B)		5.51	3.97	5.69
		(C)		2.59	1.70	2.83
		X-ray		4.43	3.43	4.65
		NMR		4.25	2.84	4.41
		All		4.37	3.24	4.57

**Table 4.** The ranking results of Rosetta, RASP-ALL, KB and 3dRNAscore methods on the FARFAR decoy set

Motif name	DI of the lowest energy model				Minimum DI model
	ROSETTAmin	RASP-ALL	RNA KB	3dRNAscore	
G-A base pair	4.026	1.408	2.455	1.408	1.408
Fragment with G/G and G/A pairs, SRP helix VI	13.910	6.073	6.073	6.073	5.267
Helix with A/C base pairs	2.414	3.262	5.410	5.866	2.414
Four-way junction, HCV IRES	21.236	21.236	21.526	20.067	15.040
Loop 8, A-type Ribonuclease P	8.421	4.290	4.290	4.290	1.615
Helix with U/C base pairs	6.012	3.136	3.136	3.136	3.136
Curved helix with G/A and A/A base pairs	1.062	1.929	3.565	1.929	0.998
Pre-catalytic conformation, hammerhead ribozyme	30.097	12.830	12.830	12.830	12.830
Loop E motif, 5S RNA	2.404	2.561	2.561	2.561	1.986
UUCG tetraloop	1.284	1.278	1.403	1.278	1.278
Rev response element high affinity site	7.802	5.826	4.227	5.826	4.227
Fragment with A/C pairs, SRP helix VI	3.930	2.424	2.424	2.424	2.424
Signal recognition particle Domain IV	5.516	3.224	3.224	3.224	1.338
Bulged G motif, sarcin/ricin loop	1.902	7.066	7.838	7.066	1.588
Tertiary interaction, hammerhead ribozyme	25.215	24.647	24.647	24.647	19.453
GAGA tetraloop from sarcin/ricin loop	1.075	0.956	0.956	0.956	0.956
Pentaloop from conserved region of SARS genome	4.147	4.145	4.147	4.145	1.068
L2/L3 tertiary interaction, purine riboswitch	16.371	19.160	19.160	19.160	16.371
L3, thiamine pyrophosphate riboswitch	6.076	2.262	2.262	2.262	2.262
Kink-turn motif from SAM-I riboswitch	1.592	13.086	12.107	12.107	1.454
Active site, hammerhead ribozyme	20.394	23.413	22.927	22.927	17.947
P1/L3, SAM-II riboswitch	14.885	18.103	10.478	18.174	10.478
J4/5 from P4-P6 domain, Tetrahymena ribozyme	2.504	2.919	2.919	2.919	2.199
Stem C internal loop, L1 ligase	3.479	3.479	3.226	3.479	3.226
J5/5a hinge, P4–P6 domain, Tetr. ribozyme	25.063	23.920	28.689	23.920	20.910
Three-way junction, purine riboswitch	13.693	13.734	10.616	10.616	10.387
J4a/4b region, metal-sensing riboswitch	6.237	5.012	6.549	5.012	5.012
Kink-turn motif	15.0735	23.997	28.729	16.805	11.695
Tetraloop/helix interaction, L1 ligase crystal	1.398	0.948	1.398	0.948	0.948
Hook-turn motif	7.598	16.240	15.354	1.985	1.985
Tetraloop/receptor, P4-P6 domain, Tetr. ribozyme	16.803	11.112	4.477	4.477	3.818
Pseudoknot, domain III, CPV IRES	10.097	7.063	7.771	4.377	4.274

### Contribution of the dihedral-dependent potential

Most statistical potentials for RNA structures are based on distance distribution of intermolecular atom pairs or residue pairs. 3dRNAscore incorporated an additional energy contribution based on the distribution of backbone dihedrals (torsion angles) in the light of molecular force field. This will be discussed in the ‘Discussion’ section.

To verify the contribution of the dihedral-based energy, we tested the performance of single distance-dependent energy, single dihedral-dependent energy and combined total energy according to Equation (12). The results are shown in Table 3. On average, single distance-dependent energy performs better than single dihedral-dependent energy but in some case (5 out of 40) the later is better than the former, especially four of the five (1a4d, 1j6s, 1l2x, and 28sp) is for FARNA dataset that consists of many near-native tertiary models (11) and they are just the cases where single distance-dependent energy performs worth than the best results of other methods (see Table 2). Furthermore, the average ES given by single distance-based energy is 4.37 while that given by the combined energy is 4.57. These results indicate that including dihedral-dependent energy can further improves accuracy of the distance-dependent energy on average.

### Relationship between 3dRNAscore and physical interactions in RNA

**Figure 5.**
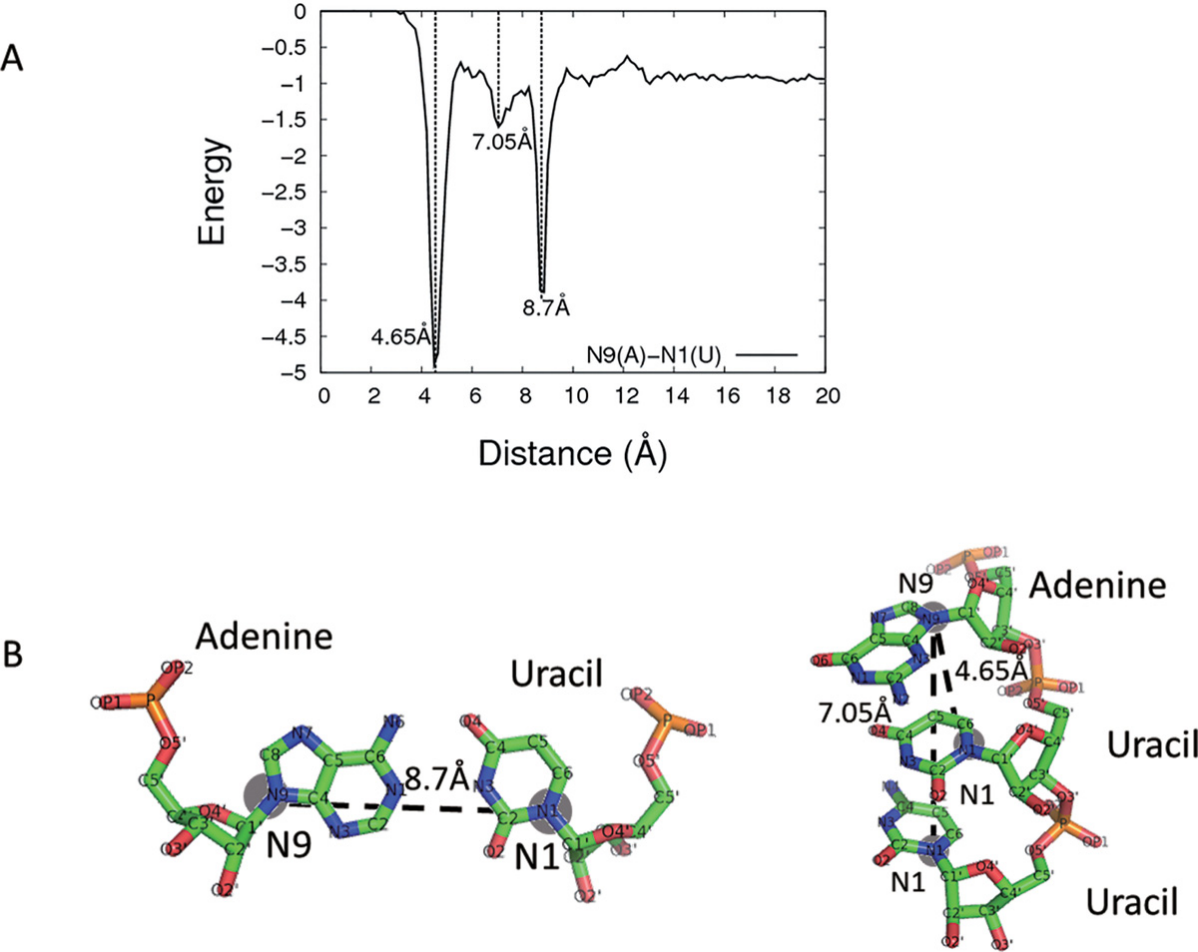
(**A**) Energy distribution of the distance between N9 of adenine and N1 of uracil. (**B**) Diagram of the three representative distance between N9 of adenine and N1 of uracil.

Capriotti and coworkers have found that all-atom version of RASP, RASP-ALL, could well capture base-pairing and base-stacking interactions in RNA. We observed the same phenomenon using 3dRNAscore. It seems that base-pairing and base-stacking interactions are implicitly contained in all-atom distance-dependent statistical potential. For example, Figure 5 depicts the distance distribution of the atom pair between N9 of adenine and N1 of uracil. Three apparent peaks appear on the distance distribution. The first peak is at the distance of 4.65 Å, and it stems from the base-stacking interaction between adjacent residues along the nucleotide residues chain. The second peak is at the distance of 7.05 Å, and it's from the indirect interaction between the *i*th residue and the (*i* + 2)th residue. The third peak is at the distance of 8.7 Å, and it results from the base-pairing interaction between adenine and uracil.

**Figure 6.**
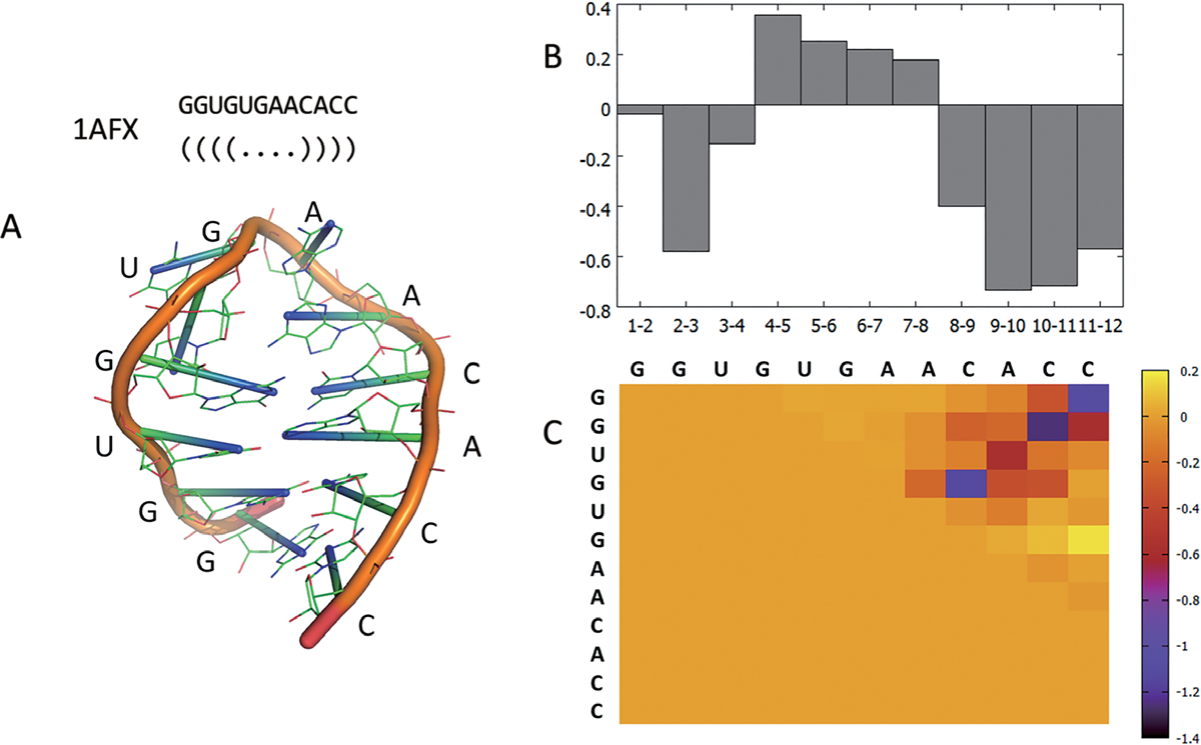
(**A**) Sequence, secondary structure and 3D structure of 1AFX. (**B**) Base-stacking energies between adjacent two nucleotides in 1AFX calculated by 3dRNAscore. ‘1–2′ means base-stacking energy between the first and the second nucleotide, ‘2–3′ means the second to the third … and so on. The lower the energy, the better the base-stacking. (**C**) Base-pairing energies between each possible base-pair in 1AFX calculated by 3dRNAscore. The lower the energy, the better the base-pairing.

Figure 6 shows the base-stacking and base-pairing energies analyzed by 3dRNAscore in RNA 1AFX, which has a hairpin 3D structure. Figure 6(B) suggests that stacking energies between the bases from the fourth to eighth nucleotides are lower than the other base-stacking energies, which just meet the 2D structure: ((((….)))). Figure 6(C) suggests that the base-pairing energies between the nucleotides 1–12, 2–11, 3–10 and 4–9 are the four lowest energies, which exactly represent the four Watson–Crick base-pairing in 1AFX.

### Choosing a structure model in a robust way

**Figure 7.**
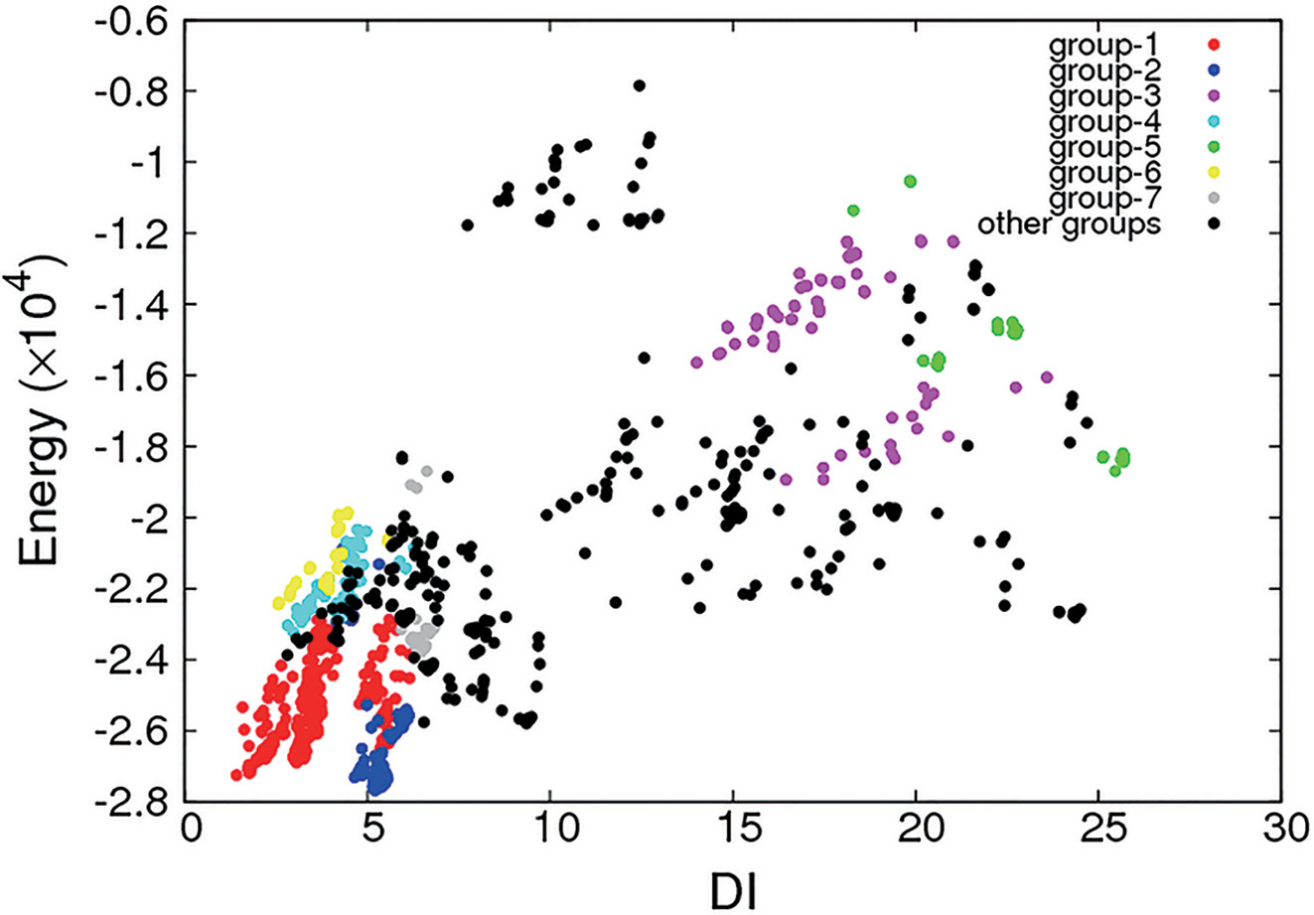
Energy versus DI plot of the groups of the 1000 prediction models of 1FQZ.

Evaluation function is used to select proper structure models. Here, we give an example of how to choose a structure model in a robust way during RNA tertiary structure prediction. We firstly use the RNA 3D structure building program 3dRNA (12) to predict 1000 models for a duplex RNA (PDB id: 1FQZ). The 1000 models can be clustered into groups based on their INF values and with a threshold. Table 5 and Figure 7 are results for a threshold of 0.18. Each group is then scored by 3dRNAscore. The structure with the lowest energy score is selected as the representative structure of its group. All of these representative structures constitute the candidates of the native structure.

**Table 5.** Seven largest groups clustered from 1000 models predicted by 3dRNA for 1FQZ

	Group I	Group II	Group III	Group IV	Group V	Group VI	Group VII	Other
Size	190	39	56	40	19	38	21	597
Center DI	3.738	5.234	18.727	4.632	23.136	4.173	6.724	13.741
Minimum DI	1.429	4.373	13.270	2.713	9.758	2.437	5.129	2.612
DI with minimum energy	1.429	4.487	16.132	2.713	26.532	2.437	5.384	9.537

Table 5 lists the seven largest groups. Figure 7 shows that Group I includes the model with the lowest DI. Although the overall energy scores of Group II are lower than Group I, the model with the lowest DI in Group I can still be picked up through clustering. Furthermore, most groups exhibit a funnel shape after clustering, which is conductive to 3dRNAscore that the model with the lowest energy score is just the one with the minimum DI.

## DISCUSSION

The native structure tends to have the lowest free energy according to the thermodynamic hypothesis proposed by Anfinsen (28). So precisely to say, we need to use free energy to evaluate a structure. For classical molecular force fields, it is easy to calculate the enthalpy of a structure but it is very time consuming to calculate its entropy. In contrast to force fields, statistical potentials extracted from experimental data of known RNA structures include both enthalpy and entropy information. Although they are not directly equal to free energy, they in principle correlate with the latter. Furthermore, the calculation is easy and fast. So, statistical potentials are more often used for RNA and protein structure scoring than molecular force fields.

A significant difference between 3dRNAscore and other RNA statistical potentials is the combination of the conventional distance-dependent energy with a dihedral-dependent energy. Thus, in principle, 3dRNAscore can take account of both overall shape and base pairs. The results above indicate a better performance of this combined potential. It is known that molecular force fields are the basis of molecular dynamics simulation to study conformations of biomolecules. Many force fields in use today consist of four components: bond stretching, angle bending, the rotation of bonds and non-bonded interactions. The distance-dependent statistical potential corresponds to non-bonded interactions part of a force field while the dihedral-dependent potential corresponds to bond rotation part. The latter can efficiently describe the flexibility of RNA molecules, which is one of the major features of RNA structures that are different from that of protein structures. Originally, we planned to design four different statistical potentials corresponding to the four components of a force field. However, we found that the potentials corresponding to bond stretching and angle bending have no significant effect because all of the decoy sets don't involve any variation of bond length and bond angle in the process of their generations.

RNA backbone is rotameric (52,53), and this may help recognize native-like models. Murray and coworkers (52) processed the backbone torsion angle distributions of an 8636-residues RNA database with quality-filtering techniques like resolution, crystallographic B factor and all-atom steric clashes. With noise levels greatly reduced, clear signal appears for the underlying angle preferences. It suggests that native-like models have obvious preference for certain specific backbone torsion angle distribution. The dihedral-dependent energy of 3dRNAscore utilizes this preference to identify the native-like model from decoys where the dihedral distributions of RNA structures deviate from the normal dihedral distributions.

The Rosetta RNA scoring function (FARFAR) (6) is more detailed and precise than most other RNA knowledge-based potentials. The RNA energy function used in FARFAR includes a term weakly favoring compactness (proportional to radius-of-gyration), a term to penalize steric clashes within molecules and other terms that are specially designed for RNA interactions (6,11). On account of these precise energy terms contained in FARFAR, its scoring performance depends largely on the quality of the decoy sets. The less good scoring performance of FARFAR than that of other three scoring methods in the test above may be attributed to its unfitness to these decoys sets.

Test set I is generated by Gaussians on distances and torsions and Test set II is generated by molecular dynamics in a relative short period of time, hence decoys in these two datasets have low diversity and are distributed around a local minimum free energy state (in these two sets, the local minimum free energy state is just the native state) in the sense of free energy landscape. Results in these two sets show that 3dRNAscore is quite qualified for identifying local minimum free energy state and ranking structures around the local minimum. Test set III is a more real-world decoy set which is generated by RNA modelling with FARFAR (6), so decoys in test set III have a very high diversity, which is owed to the Monte Carlo algorithm adopted by FARFAR. Results in test set III show that 3dRNAscore's performance in ranking structures widely distributed in free energy landscape is not so good, but still better than other existing statistical potentials.

Other limitations exist for knowledge-based statistical potentials at present. The limited number of RNA tertiary structures of the training set is still the major problem for developing knowledge-based potentials (54–56). Almost all the knowledge-based potentials face the possibility of over-training problem because the parameters depend on the limited number of structures in training datasets (57). We have tried to use a non-redundant RNA tertiary structure set as the training set and then remove all the structures that are similar to certain structures in test sets. More non-redundant structures would improve the accuracy of knowledge-based potentials (58,59). Furthermore, RNAs are very sensitive to electrostatic interactions because of the negative charges in the phosphate groups of the backbone (60–62). RNA may be unable to form the functional folds in the absence of positive ions. The knowledge-based potentials only implicitly consider this effect by counting the experimental structures. We will study these problems in future.

## CONCLUSION

In this paper, we have developed a novel RNA knowledge-based potential for identifying native RNA structures and ranking predicted structures and functional structural motifs with non-canonical base pairs. We used a non-redundant RNA training set to train the parameters by combining the distances of paired atoms and torsion angles to construct our statistical potential. The benchmark tests show that our method could identify not only appropriate tertiary folds, but also tertiary motifs with the non-canonical base pairs. Although some limitations, 3dRNAscore performs consistently better than existing methods in both two cases.

## AVAILABILITY

http://biophy.hust.edu.cn/download.html.

## SUPPLEMENTARY DATA

Supplementary Data are available at NAR Online.

SUPPLEMENTARY DATA
