# Supplementary material for: 3dRNAscore: a distance and torsion angle dependent evaluation function of 3D RNA structures
Source: Nucleic Acids Res. 2015 Feb 24;43(10):e63. doi: 10.1093/nar/gkv141 (PMC4446410; doi:10.1093/nar/gkv141)
Supplement: SUPPLEMENTARY DATA [file supp_43_10_e63__index.html]

3dRNAscore: a distance and torsion angle dependent evaluation function of 3D RNA structures — SUPPLEMENTARY DATA 

# 3dRNAscore: a distance and torsion angle dependent evaluation function of 3D RNA structures

## SUPPLEMENTARY DATA

**Files in this Data Supplement:**

- SUPPLEMENTARY DATA
