# Supplementary material for: 3dRNAscore: a distance and torsion angle dependent evaluation function of 3D RNA structures
Source: Nucleic Acids Res. 2015 Feb 24;43(10):e63. doi: 10.1093/nar/gkv141 (PMC4446410; doi:10.1093/nar/gkv141)
Supplement: SUPPLEMENTARY DATA [file supp_gkv141_nar-03468-met-z-2014-File009.pdf]

Supplementary material for
3dRNAscore: a distance and torsion angle dependent evaluation function of three-dimensional RNA structures

Jian Wang*, Yunjie Zhao*, Chunyan Zhu and Yi Xiao†

Biomolecular Physics and Modeling Group, Department of Physics and Key Laboratory of Molecular Biophysics of the Ministry of Education, Huazhong University of Science and Technology, Wuhan 430074, Hubei, China

*These authors made equal contributions.
†Corresponding author (email: yxiao@hust.edu.cn).

Contents

1 Comparison of the performance of 3dRNAscore, KB potential, RASP, and Rosetta methods in test set II 3

2 Energy-RMSD plots of the results in test set II 5

3 Energy-DI plots of the results in test set II 7

4 Energy-INF$_{all}$ plots of the results in test set II 9

5 Energy-INF$_{wc}$ plots of the results in test set II 11

6 Energy-INF$_{stack}$ plots of the results in test set II 13

1 Comparison of the performance of 3dRNAscore, KB potential, RASP, and Rosetta methods in test set II

Decoy	RNA	Length	Method	ES(RMSD)				ES(DI)				ES(INF_{all})				ES(INF_{wc})				ES(INF_{stack})			
				D[1]	K[2]	R[3]	S[4]	D	K	R	S	D	K	R	S	D	K	R	S	D	K	R	S
Position restrained dynamics and REMD (A)	1duq	26	X-ray	8.5	7.6	7.6	7.1	8.3	7.5	7.6	7	9.4	8.5	8.3	7.1	9	8.3	8	6.9	8.7	7.8	8	6.9
	1f27	30	X-ray	8.3	7.9	6.6	6.2	8.1	7.8	6.6	6.2	8.2	7.9	8.2	6.3	8.2	7.9	7.8	6.1	7.6	7.8	7.9	5.9
	1msy	27	X-ray	7.5	5.7	5.7	3.6	7.6	6	5.6	3.5	6.8	7.3	6.7	3.7	4	3.8	3.8	2.4	6.3	5.6	5.8	3.4
	1nuj	24	X-ray	7.7	7.3	5.2	6.9	7.4	7.2	5.2	6.7	8.5	7.7	7.7	6.4	8.8	7.3	6.5	5.3	7.1	7.3	7.7	6.4
	434d	14	X-ray	8	7.7	7	6.8	7.6	7.7	6.9	6.8	8.7	8.1	8.5	6.9	8.7	8.5	8.2	6.5	8.4	7.2	7.4	6.3
Normal modes (B)	1duq	26	X-ray	7.5	7	5.7	3.8	6.9	7	5.7	3.5	2.4	2.3	2.9	3.8	4.3	2.9	3.3	3.8	3.7	3.7	3.7	4.3
	1esy	19	NMR	4.9	5.4	4.5	5.6	6.5	5.5	4.7	5.7	2.6	1.5	2.4	1.8	0.7	0.2	0.4	0.2	2.2	1.8	2	1.5
	1f27	30	X-ray	5.9	5.8	3.7	2.6	6.7	5.8	3.7	2.5	3.9	3.4	2.9	2.3	3.3	2.9	2.2	1.7	4	2.6	3.7	2.8
	1i9v	76	X-ray	6.1	2.6	5.3	3	5.5	2.7	5.1	3	5.5	5.1	4.7	2.8	5.7	3.9	4.1	2.5	3.6	2.9	3.3	1.9
	1kka	17	NMR	5.7	4.6	4.1	4.6	6.9	4.3	4.1	4.8	3.5	2.9	2.9	3.2	0.3	0.2	0.2	0.3	3.2	2.5	2.2	2.8
	1msy	27	X-ray	5.9	5.6	2.2	4.6	6.7	5.4	2.7	4.5	4.2	3.3	3.7	3.3	0.4	0.5	1	0.8	3.6	2.3	3.5	3.3
	1nuj	24	X-ray	7.1	7.4	5.9	2.4	4.5	7	5.9	2.2	3.5	3.2	3.3	2.1	4.1	3.2	3.9	3.2	3.3	2.2	2.4	1.9
	1qwa	21	NMR	3.5	3.2	2	3.8	3.7	3.3	2.2	3.9	3.1	2.1	2.6	2.7	2.9	2.3	2.6	2.7	2.7	1.4	1.8	2.2
	1x9k	62	X-ray	6.7	1.6	5.2	3	3.1	1.9	5.2	2.8	5.6	4.9	4.6	2.8	5	4.9	4.6	2.6	5	4.8	4	2.5
	1xjr	46	X-ray	7.7	5.4	7.9	2.2	6.2	5	7.9	2.5	6	5.2	5.4	2.3	4.4	4.3	4.8	1.8	5.2	3.9	4.6	2.1
	1ykq	19	X-ray	4.6	3.4	3.5	2.8	5	3.3	3.8	2.5	6	5.5	4.8	2.4	5	3.8	3.7	2.6	4.4	4.3	4.1	1.7
	1zih	12	NMR	7.7	5.4	5.7	6.6	6.9	5.3	5.7	6.5	2.6	2.6	2.6	2.3	0	0	0	0	3.4	3.8	3.5	3.3
	28sp	28	NMR	5.7	4	6.5	1.8	5.7	4.5	6.7	2.9	5.7	5.2	4.9	2.5	3.3	2.7	2.6	1.2	3.9	3.3	4.7	2.4
	2f88	34	NMR	6.8	5.4	4.9	4.4	4.1	5.4	4.7	4.2	4.1	4	4.1	3.2	5.7	5.1	4.9	3.3	3.1	1.8	2.3	1.8
	434d	14	X-ray	7.7	7.4	7.4	5.2	6.9	7.4	7.6	5.2	4.3	3.8	3.5	3.6	3.9	2.9	2.6	2.7	4.3	3.4	3.5	3.6
FARNA (C)	1a4d	41	NMR	2.4	3.8	2	0.8	2.1	4	2	0.7	3	3.2	3	0.4	1.8	1.3	1.2	0.2	3.4	2	2	0.4
	1csl	28	X-ray	2	1.5	1.6	1.3	2.4	1.4	1.6	1.4	2.4	2.7	3.2	1.3	2	2.7	2.4	0.8	2.4	2.5	2.6	1.1
	1dqf	19	X-ray	4.2	1.8	2.8	1	3.6	2	3.2	1	2.4	3.4	3.2	1	3	2.1	2.6	0.9	2.8	3	3	0.9
	1esy	19	NMR	3.2	3.7	4.8	1.2	3.8	3.5	4.2	1.1	3.4	2.5	2.2	1.3	4.2	3.2	1.8	1.1	2.8	1.7	2.6	1.5
	1i9x	26	X-ray	3.4	1.3	2.2	1.5	4	1.5	3.6	1.6	3	2.8	2.2	1.7	3	2.5	2.4	1.7	2	2	2	1.6
	1j6s	24	X-ray	0.2	1.4	0.2	0.6	1.6	1.7	0.8	0.5	3.4	3.1	3	0.5	1.2	0.9	0.6	0.4	4.8	4.3	4	1
	1kd5	22	X-ray	3.2	0.3	0.8	0.2	2.4	0.5	1.6	0.3	2.6	2.9	2.6	0.2	2	1.2	1.6	0.3	4.2	3.3	3.8	0.8
	1kka	17	NMR	1.4	1.2	0.6	0.6	2	0.8	0.8	0.6	3	2.5	3.4	2.1	3.4	2.7	1.8	1.4	2.8	2.8	3	1.8
	1l2x	27	X-ray	0.6	3.2	1	1.8	1.5	3.2	1.2	1.8	2.4	2	1.8	1.8	0.4	0.1	0	0.3	3	2.4	2.2	1.8
	1mhk	32	X-ray	1.6	1.2	1.6	1	1.4	1.2	1.4	1.2	2.6	1.9	2.2	1.2	2.2	2.5	2.6	1.2	2	1.7	1.8	1.2
	1q9a	27	X-ray	2.6	0.5	0.8	0.8	3.2	0.5	1.2	0.8	2	1.8	1.6	0.8	0.8	1	1	0.6	3.8	3.1	3.2	1.5
	1qwa	21	NMR	2.2	1.2	0.4	1	1.4	1.3	0.6	1	2.4	2.7	2.6	1	1.8	1.8	1.8	1.1	3.2	2.9	2.6	1.1

Decoy	RNA	Length	Method	ES(RMSD)				ES(DI)				ES(INF_{all})				ES(INF_{wc})				ES(INF_{stack})			
				D[1]	K[2]	R[3]	S[4]	D	K	R	S	D	K	R	S	D	K	R	S	D	K	R	S
	1xjr	46	X-ray	2.4	2	2.4	1.2	3.2	1.9	3.4	1	3.8	4.2	4.4	1.2	2.2	1.6	1.8	0.5	3.6	3.6	3.6	0.9
	1zih	12	NMR	4.8	5	4.8	2	6.8	5.5	7.2	1.9	4.6	2.1	1.6	2	3.6	1.6	0.4	0.7	2.6	2.8	3.8	2.9
	255d	24	X-ray	2.4	0.7	0.6	1.3	2	0.7	0.6	1.1	2.2	2.3	1.8	1.2	1.8	1.3	1	0.9	2	1.4	1.4	1
	283d	24	X-ray	1.4	0.8	0.8	0.7	1.8	0.8	1	0.7	2.8	2.5	3	0.8	2.6	2.3	0.8	0.5	3.1	2.7	2.8	0.7
	28sp	28	NMR	2.8	1.5	3	1.7	3.8	1.2	4.2	1.8	4.8	3.9	4.2	1.6	3.2	2.7	2.4	1.2	3.9	2.9	3	1.3
	2a43	26	X-ray	2.2	2	1.4	0.6	3.2	1.8	2	0.6	3	3.4	2.8	0.5	2.2	2.1	1.8	0.5	3.5	3	3.2	0.7
	2f88	34	NMR	3.2	1.3	2.2	1.3	3.6	1.3	1.6	1.2	2.6	2.7	2.2	1.2	3.8	2.3	1.8	0.8	2.7	1.4	2.2	1.2
AVERAGE VALUES		A		8.00	7.24	6.42	6.12	7.80	7.24	6.38	6.04	8.32	7.90	7.88	6.08	7.74	7.16	6.86	5.44	7.62	7.14	7.36	5.78
		B		6.23	4.95	4.97	3.76	5.69	4.92	5.05	3.78	4.20	3.67	3.69	2.74	3.27	2.65	2.73	1.96	3.71	2.98	3.29	2.54
		C		2.43	1.81	1.79	1.08	2.83	1.83	2.22	1.07	2.97	2.77	2.68	1.15	2.38	1.89	1.57	0.79	3.08	2.61	2.78	1.23
		X-Ray		4.82	3.81	3.66	2.78	4.65	3.80	3.89	2.73	4.45	4.20	4.12	2.62	3.78	3.28	3.20	2.21	4.32	3.80	3.97	2.55
		NMR		4.18	3.52	3.50	2.72	4.41	3.53	3.75	2.79	3.49	2.92	2.98	1.95	2.67	2.01	1.68	1.09	3.07	2.39	2.75	1.86
		ALL		4.61	3.71	3.61	2.76	4.57	3.71	3.84	2.75	4.13	3.77	3.74	2.39	3.41	2.86	2.69	1.84	3.91	3.33	3.56	2.32

[1] 3dRNAscore
[2] KB
[3] RASP
[4] Rosetta

2 Energy-RMSD plots of the results in test set II

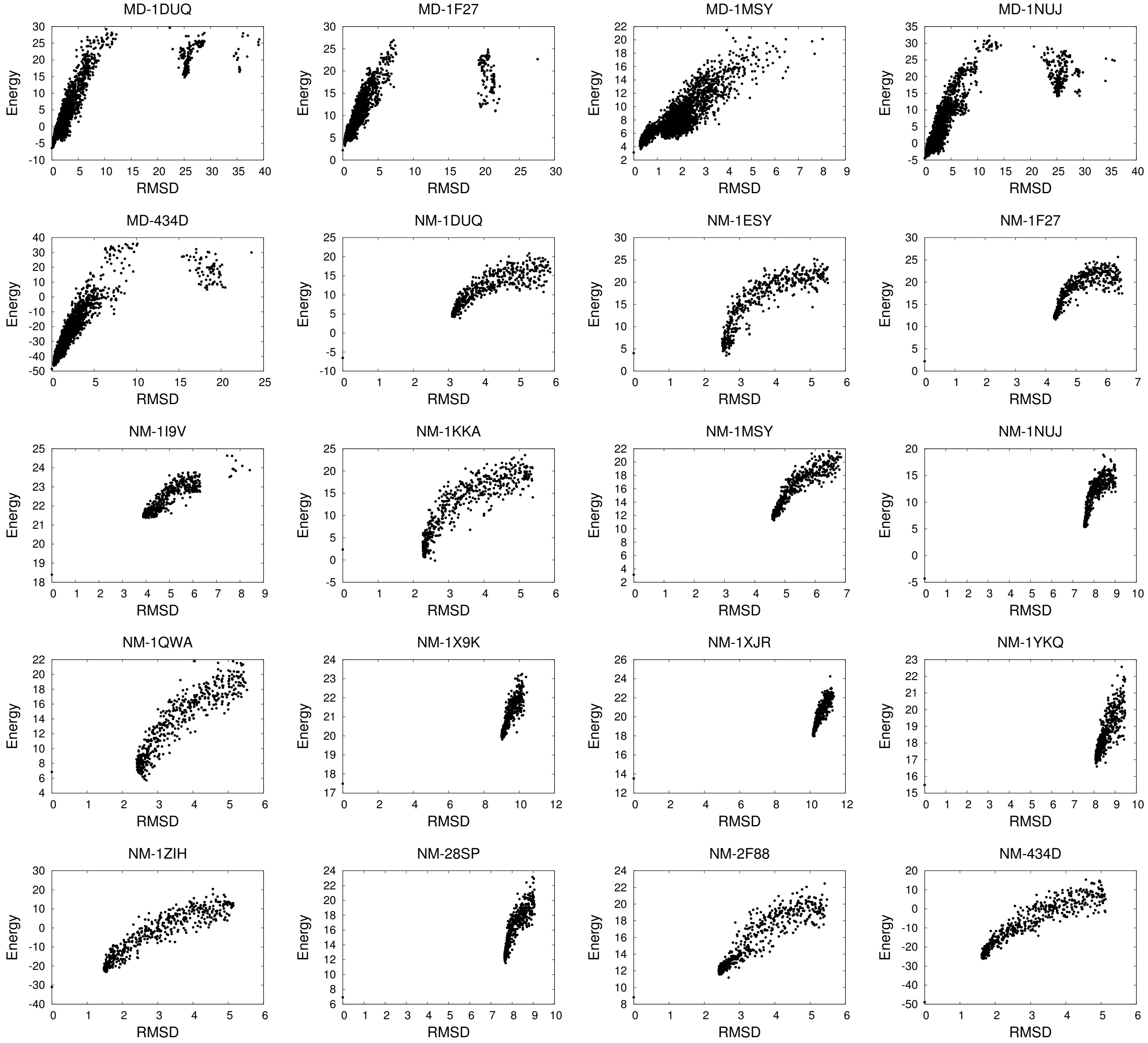

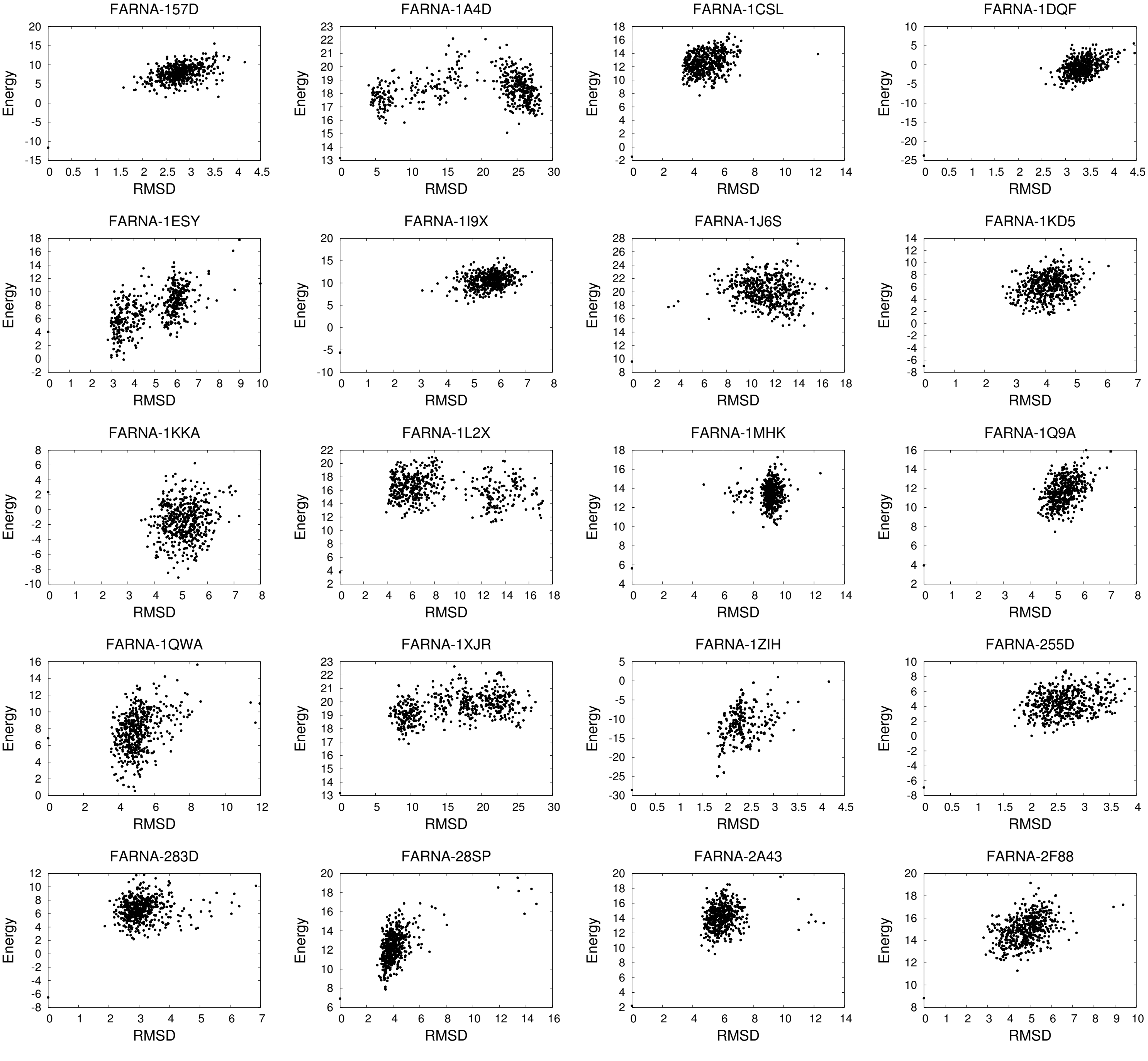
FARNA-157D
FARNA-1A4D
FARNA-1CSL
FARNA-1DQF
FARNA-1ESY
FARNA-1I9X
FARNA-1J6S
FARNA-1KD5
FARNA-1KKA
FARNA-1L2X
FARNA-1MHK
FARNA-1Q9A
FARNA-1QWA
FARNA-1XJR
FARNA-1ZIH
FARNA-255D
FARNA-283D
FARNA-28SP
FARNA-2A43
FARNA-2F88
Energy
RMSD

3 Energy-DI plots of the results in test set II

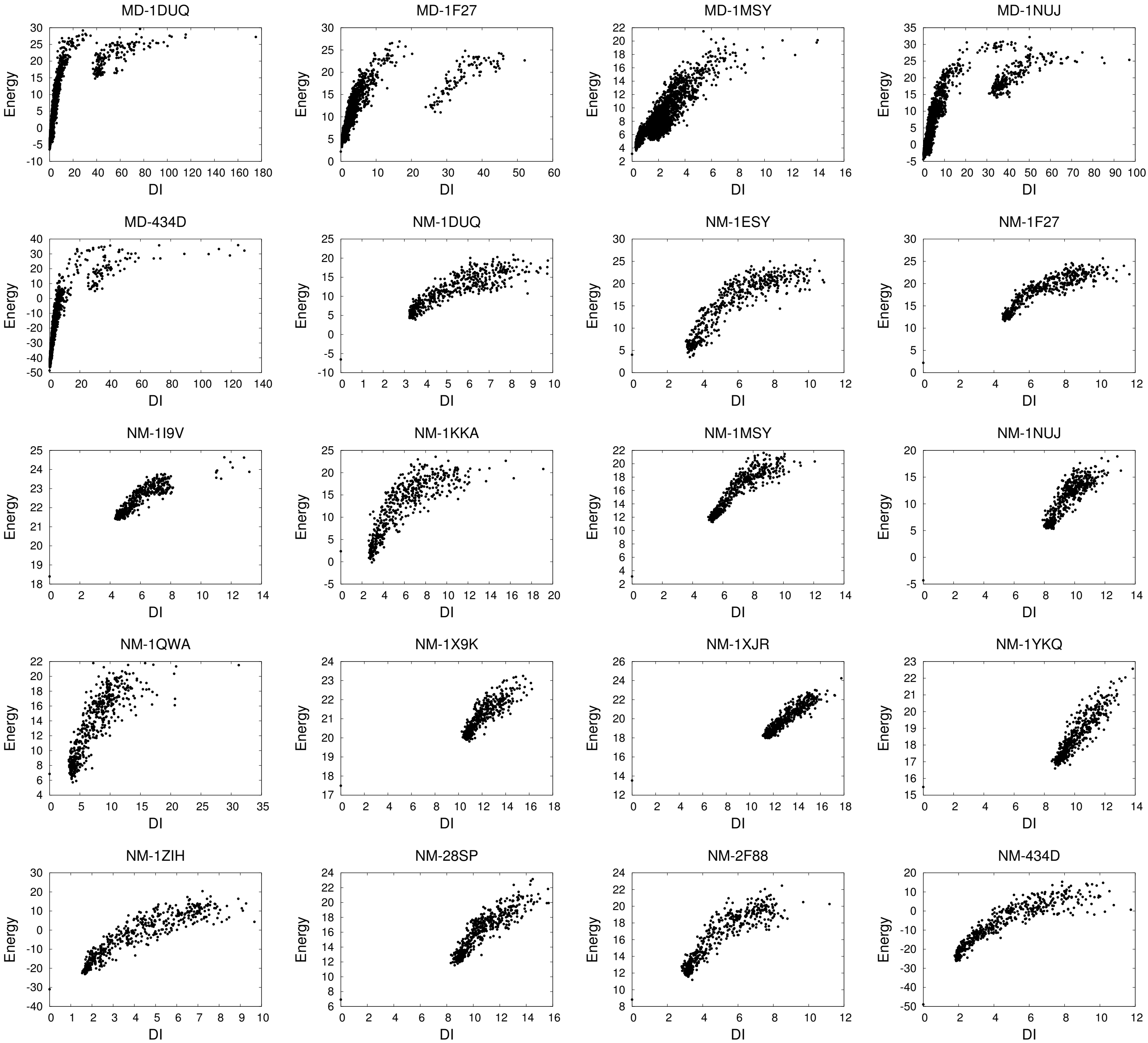

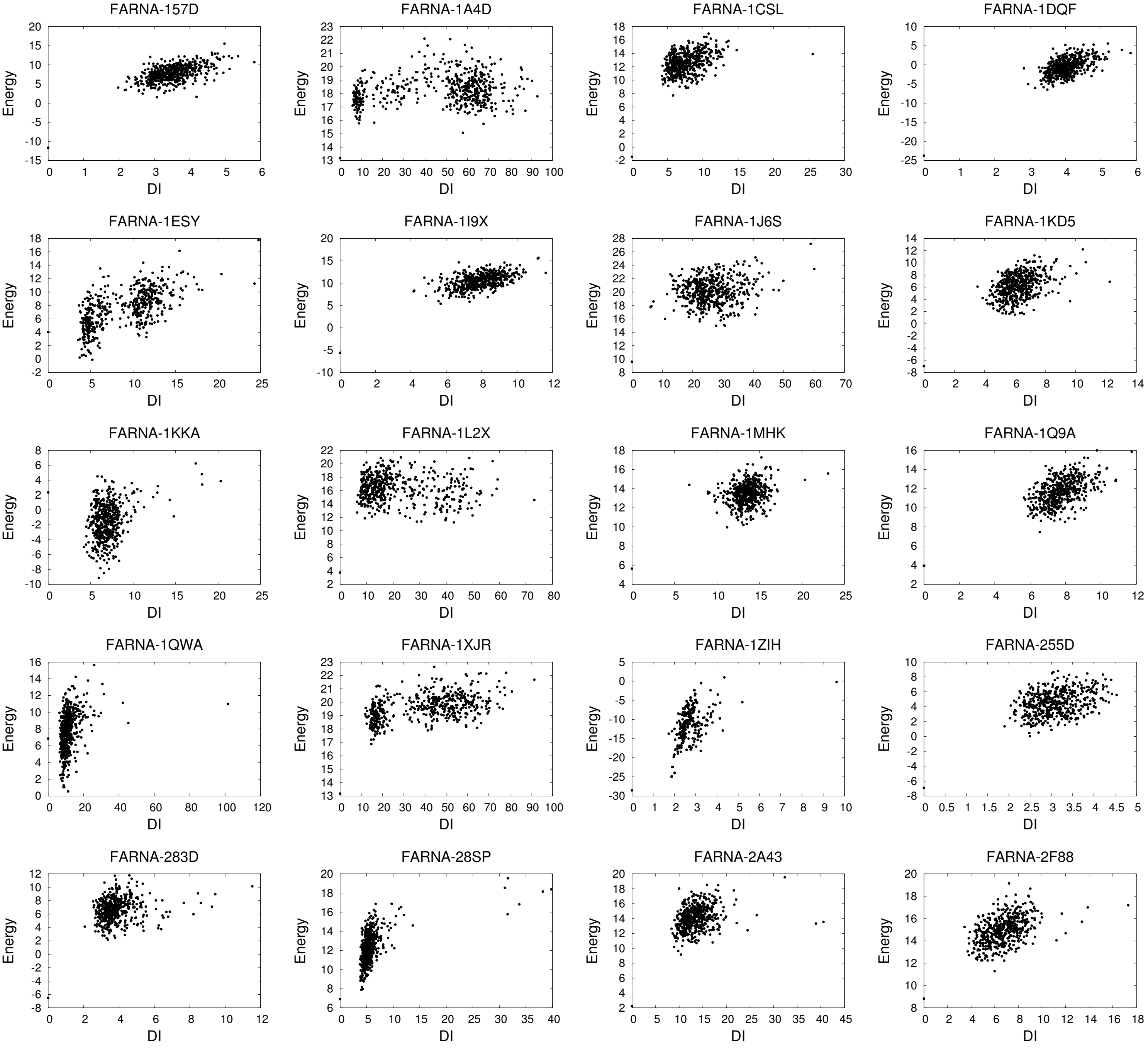
FARNA-157D
FARNA-1A4D
FARNA-1CSL
FARNA-1DQF
FARNA-1ESY
FARNA-1I9X
FARNA-1J6S
FARNA-1KD5
FARNA-1KKA
FARNA-1L2X
FARNA-1MHK
FARNA-1Q9A
FARNA-1QWA
FARNA-1XJR
FARNA-1ZIH
FARNA-255D
FARNA-283D
FARNA-28SP
FARNA-2A43
FARNA-2F88
Energy
DI

4 Energy-INF$_{all}$ plots of the results in test set II

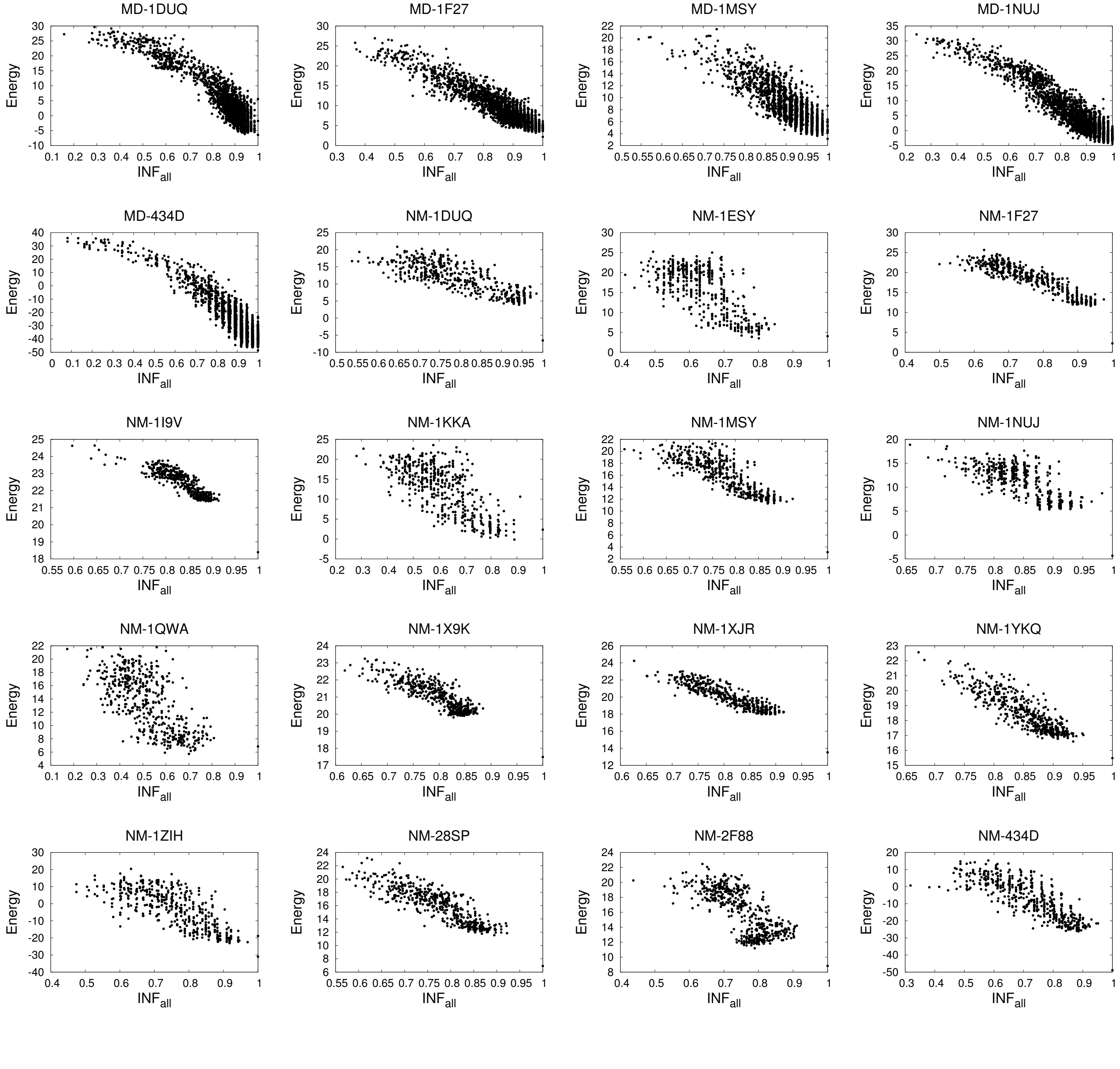

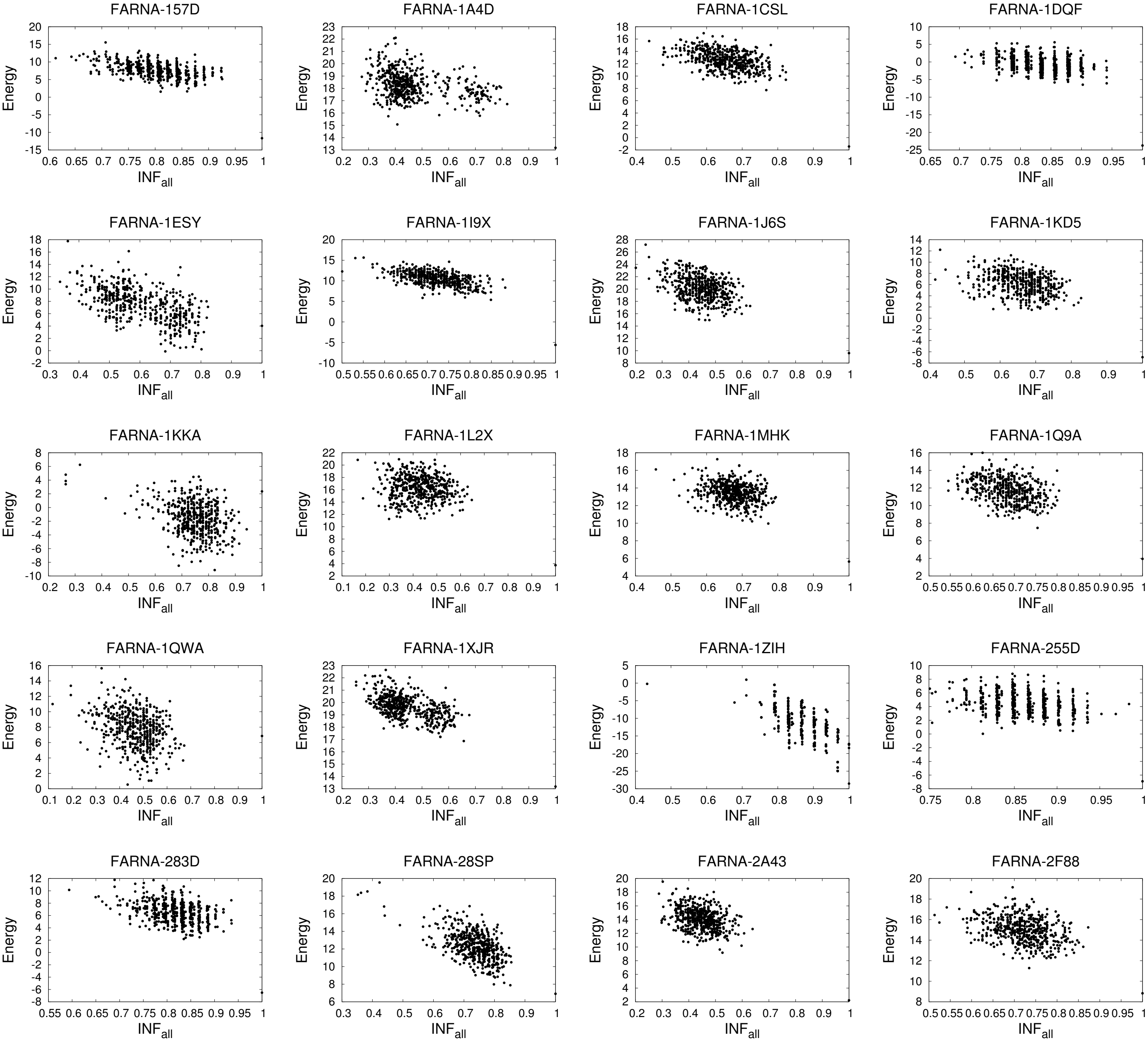
FARNA-157D
FARNA-1A4D
FARNA-1CSL
FARNA-1DQF
FARNA-1ESY
FARNA-1I9X
FARNA-1J6S
FARNA-1KD5
FARNA-1KKA
FARNA-1L2X
FARNA-1MHK
FARNA-1Q9A
FARNA-1QWA
FARNA-1XJR
FARNA-1ZIH
FARNA-255D
FARNA-283D
FARNA-28SP
FARNA-2A43
FARNA-2F88
Energy
INF$_{all}$

5 Energy-INF$_{wc}$ plots of the results in test set II

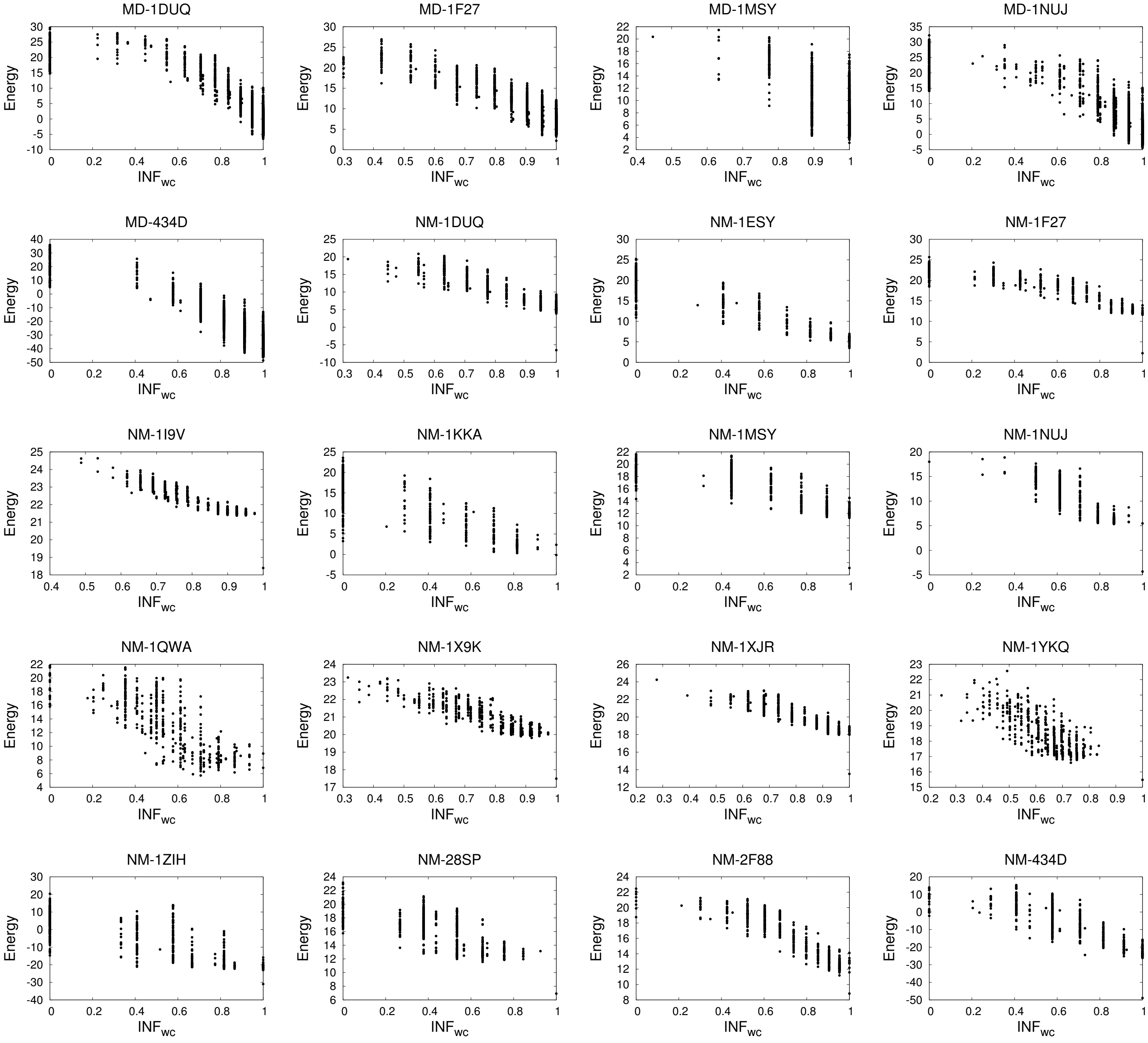

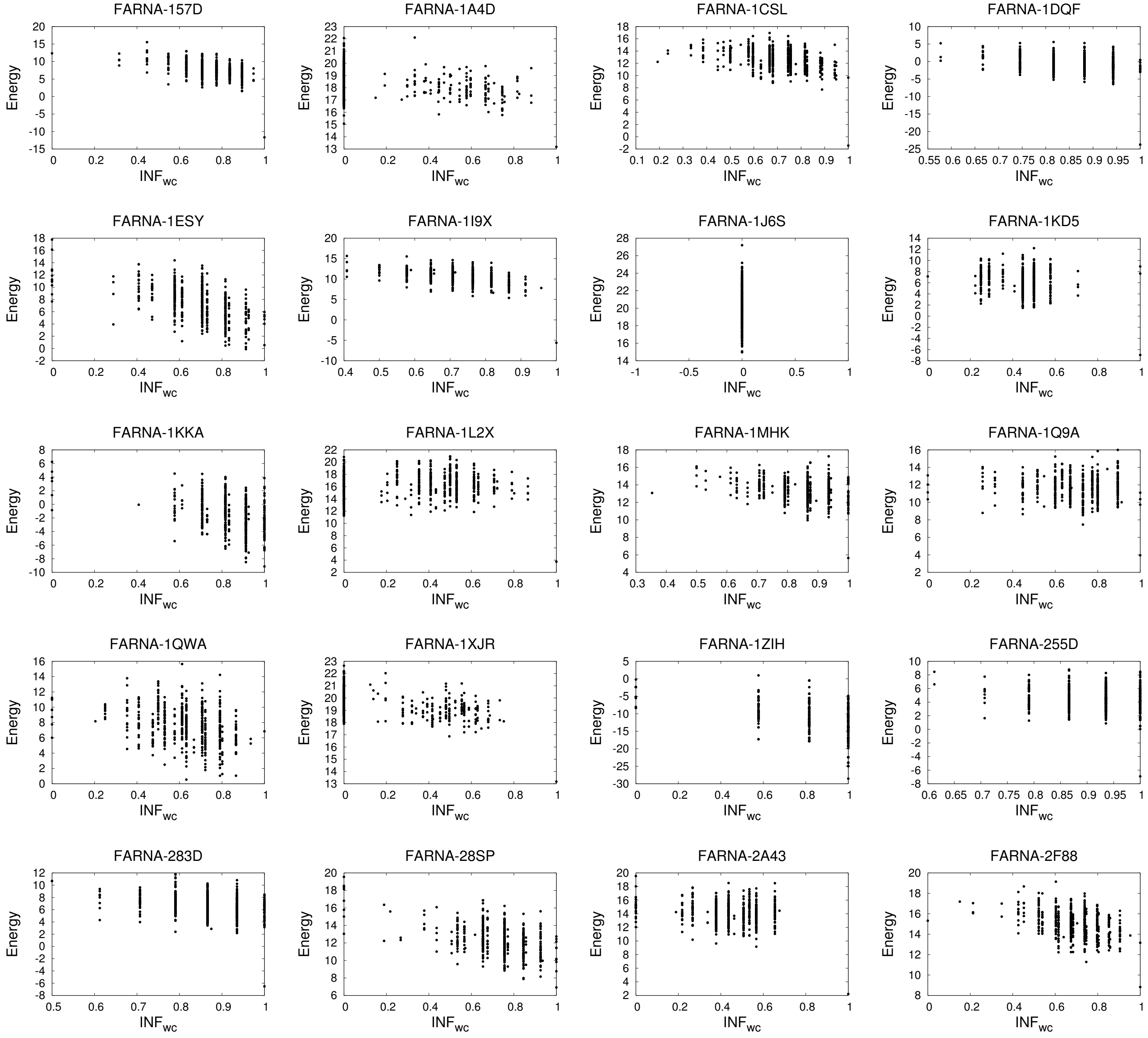
FARNA-157D
FARNA-1A4D
FARNA-1CSL
FARNA-1DQF
FARNA-1ESY
FARNA-1I9X
FARNA-1J6S
FARNA-1KD5
FARNA-1KKA
FARNA-1L2X
FARNA-1MHK
FARNA-1Q9A
FARNA-1QWA
FARNA-1XJR
FARNA-1ZIH
FARNA-255D
FARNA-283D
FARNA-28SP
FARNA-2A43
FARNA-2F88
Energy
INF_{wc}

6 Energy-INF$_{stack}$ plots of the results in test set II

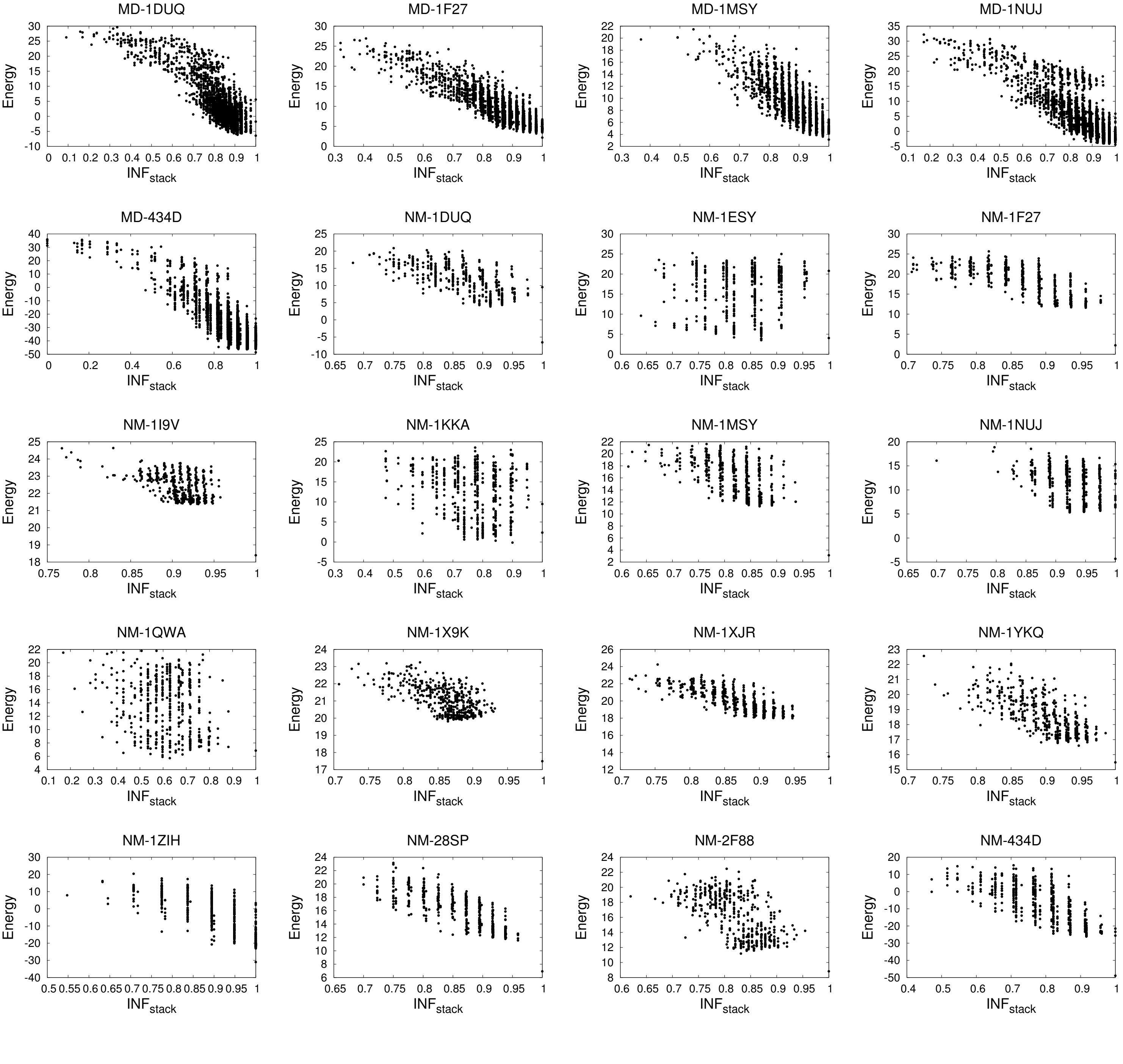

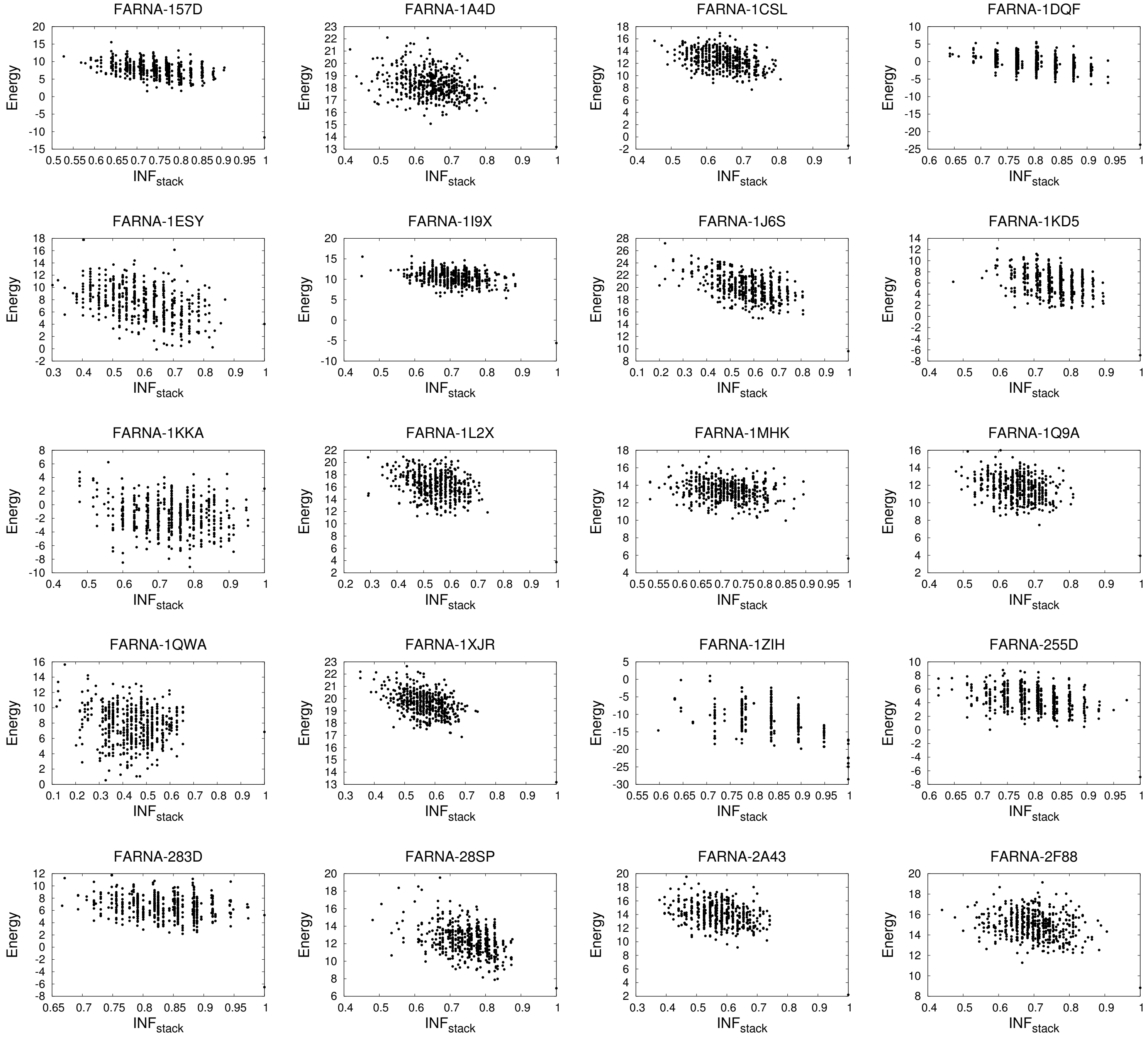
FARNA-157D
FARNA-1A4D
FARNA-1CSL
FARNA-1DQF
FARNA-1ESY
FARNA-1I9X
FARNA-1J6S
FARNA-1KD5
FARNA-1KKA
FARNA-1L2X
FARNA-1MHK
FARNA-1Q9A
FARNA-1QWA
FARNA-1XJR
FARNA-1ZIH
FARNA-255D
FARNA-283D
FARNA-28SP
FARNA-2A43
FARNA-2F88
Energy
INF$_{stack}$
